# Tailoring the Reversible Phase Transition of Perovskite Nanofiber Electrodes for High-Performance and Durable Reversible Solid Oxide Cells

**DOI:** 10.1007/s40820-024-01600-4

**Published:** 2025-02-17

**Authors:** Chaofan Yin, Jiaming Yang, Jiangyuan Feng, Yueyue Sun, Zhengrong Liu, Junkai Wang, Jiajia Cui, Zixuan Xue, Liang Zhang, Yucun Zhou, Jun Zhou, Liangfei Xu, Kai Wu, Jianqiu Li

**Affiliations:** 1 Center of Nanomaterials for Renewable Energy, State Key Laboratory of Electrical Insulation and Power Equipment, Xi’an Jiaotong University, Xi’an, 710049 People’s Republic of China; 2 Beijing Huairou Laboratory, Beijing, 101400 People’s Republic of China; 3 Xi’an Thermal Power Research Institute Co., Ltd, Xi’an, 710054 People’s Republic of China; 4 School of Materials Science and Engineering, Xi’an University of Technology, Xi’an, 710048 People’s Republic of China; 5 School of Vehicle and Mobility, Tsinghua University, Beijing, 100084 People’s Republic of China

**Keywords:** Reversible solid oxide cells, Reversible phase transition, Exsolution and dissolution, CO_2_ electrolysis

## Abstract

Reversible solid oxide cells (RSOCs) are capable of converting various energy resources, between electricity and chemical fuels, with high efficiency and flexibility, making them suitable for grid balancing and renewable energy consumption. However, the practical application of RSOCs is still limited by the insufficient activity and stability of the electrodes in different operating modes. Herein, a highly efficient symmetrical electrode composed of La_0.3_Sr_0.6_Ti_0.1_Co_0.2_Fe_0.7_O_3−*δ*_ (LSTCF) nanofibers and in situ exsolved Co_3_Fe_7_ nanoparticles is developed for boosting the performance of RSOCs. The reversible phase transition, high activity and stability of the electrode have been confirmed by a combination of experimental (e.g., transmission electron microscopy and X-ray absorption fine structure) and computational studies. Electrolyte-supported RSOCs with the symmetrical electrode demonstrate excellent catalytic activity and stability, achieving a high peak power density of 0.98 W cm^−2^ in the fuel cell mode using H_2_ as the fuel (or 0.53 W cm^−2^ using CH_4_ as the fuel) and a high current density of 1.09 A cm^−2^ at 1.4 V in the CO_2_ electrolysis mode (or 1.03 A cm^−2^ at 1.3 V for H_2_O electrolysis) at 800 °C while maintaining excellent durability for over 100 h.

**Supplementary Information:**

The online version contains supplementary material available at 10.1007/s40820-024-01600-4.

## Highlights

- La_0.3_Sr_0.6_Ti_0.1_Co_0.2_Fe_0.7_O_3−*δ*_ (LSTCF) nanofibers showed high structural reversibility.
- LSTCF fibers and CoFe nanoparticles were reconstructed through a reversible phase transition process.
- The LSTCF fiber electrode demonstrated excellent activity and stability for power and fuel co-generation.

**Supplementary Information:**

The online version contains supplementary material available at 10.1007/s40820-024-01600-4.

## Graphical abstract

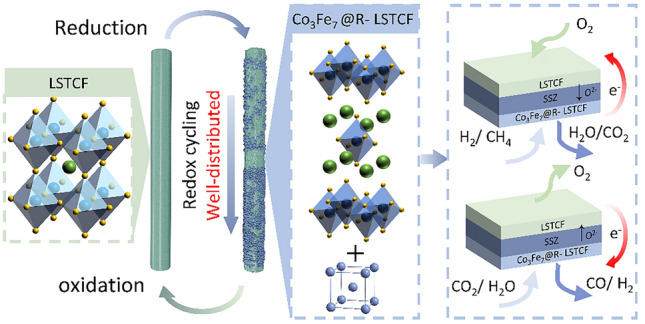

## Introduction

With the rapid increase in global energy consumption and the continuous pursuit of sustainable development, RSOCs are regarded as a promising energy conversion and storage technology due to the high efficiency, excellent reversibility, and low cost. RSOCs offer significant potential for large-scale “power-to-gas” (P2G) and “gas-to-power” (G2P) conversions, which helps to stabilize power output and manage peak loads. Consequently, RSOCs serve as a critical interconnection between the power grid and industrial network [1, 2]. Additionally, RSOCs can promote the production of energy chemicals that enhance the carbon cycle [3–5]. However, the development of RSOCs is still limited by the insufficient electrochemical activity and stability of the electrode materials [2, 4].

In situ exsolution as a kind of surface modification technology has the advantages of uniform dispersion of nanoparticles, strong coupling between exsolved nanoparticles and the matrix, and improved activity and contaminant-tolerance by the heterostructure [6–11]. Many studies have investigated the application of in situ exsolution technology to solid oxide fuel cells (SOFCs), especially fuel electrodes [12, 13]. The exsolved nanoparticles with strong coupling with the substrate enhanced the activity and stability of the fuel electrode for hydrogen oxidation reaction (HOR). In addition, the in situ exsolution technology has been applied to the fuel electrodes of solid oxide electrolysis cells (SOECs) for H_2_O and/or CO_2_ electrolysis with improved electrochemical performance and stability [13–18].

The structural stability of perovskites during the exsolution of nanoparticles in the reducing atmosphere is critical. For example, Xu et al. synthesized a Sr_2_Ti_0.8_Co_0.2_FeO_6_ electrode, in which the cubic double perovskite structure remained constant after the exsolution of Co–Fe alloy nanoparticles [13]. Similarly, Mo doping at the B-site improved the structural stability of La_0.4_Sr_0.6_Co_0.2_Fe_0.7_Mo_0.1_O_3−*δ*_ (LSCFM) under reducing atmosphere [17]. Conversely, some perovskite electrodes may undergo structural changes as the nanoparticles exsolved from the bulk. Kwon et al. found the transformation of simple perovskite Pr_0.5_Ba_0.5_Mn_0.85_T_0.15_O_3−*δ*_ (T = Mn, Co, Ni) into layered perovskite PrBaMn_1.7_T_0.3_O_5+*δ*_ (T = Mn, Co, Ni) after reduction in humidified H_2_ [19]. In addition, the conversion of ABO_3_ perovskites into Ruddlesden–Popper perovskite after reduction has been reported [20]. Luo et al. realized the control of the matrix structure during the exsolution process by adjusting the reduction time and introducing the B-site supplement mechanism [21, 22].

Flexible nanoparticle regulation and combined optimization methods are critical to the applications of in situ exsolution technology. The exsolution/redissolution strategy is effective in regulating exsolved nanoparticles. Wang et al. found that multiple redox cycles contributed to the accumulation of Ru on the surface of the perovskite matrix and increased the density of exsolved nanoparticles [23]. Sasaki et al. conducted 50 redox cycles on a Gd_0.1_Ce_0.8_Ni_0.1_O_2_-(Sr_0.9_La_0.1_)_0.9_Ti_0.9_Ni_0.1_O_3_ electrode, revealing that multiple cycles promoted the exsolution of Ni and improved the electrochemical performance. However, excessive redox cycling can lead to the aggregation of Ni [24].

RSOCs with symmetrical electrodes, i.e., RSOCs using the same material for the fuel and air electrodes, have the advantages of high reversibility, high redox stability, and low manufacturing and maintenance costs. The unique design can mitigate the effect of coking and sulfur poisoning by reversibly manipulating the gas composition in each electrode. However, the development of well-functional symmetrical RSOCs imposes higher demands on the performance of the electrode material: it needs to maintain excellent stability, conductivity, and catalytic activity in both oxidizing and reducing atmospheres [25, 26]. The La_*x*_Sr_1−*x*_TiO_3−*δ*_ (LST) perovskite material stands out for its ability to maintain a single-phase structure in wide range of oxygen partial pressure [27]. In addition, the ionic conductivity, thermal stability, and catalytic activity of LST can be improved by introducing A-site defects and doping the B-site with transition metal elements [28, 29]. Moreover, studies have demonstrated that the introduction of highly active metal nanoparticles via in situ exsolution technology could significantly improve the electrochemical performance of the LST electrode [29, 30].

When used as electrodes for RSOCs, well-designed one-dimensional (1D) materials, such as nanofibers fabricated by electrospinning, outperform particle materials due to the enhanced mass transfer process with minimized concentration polarization, larger active areas for oxygen reduction reaction (ORR) and oxygen evolution reaction (OER), better electronic and ionic conductivity, and morphological stability [31, 32]. In this study, by a combination of the benefits of the symmetrical perovskite electrode material, the nanofiber electrode structure, and the reversible phase transition approach, La_0.3_Sr_0.6_Ti_0.1_Co_0.2_Fe_0.7_O_3−*δ*_ (LSTCF) nanofiber electrodes were developed for RSOCs. The LSTCF fiber electrode with well-distributed Co_3_Fe_7_ nanoparticles reconstructed by the exsolution/redissolution strategy demonstrated excellent catalytic activity and stability for power generation, H_2_O electrolysis, and CO_2_ electrolysis. The comprehensive investigation of the perovskite material’s redox behavior, as well as the established reconstruction technique, will advance the understanding and application of this type of electrode materials for high-performance and durable RSOCs.

## Experimental Section

### Materials Synthesis

The LSTCF fiber was prepared by an electrostatic spinning method. The process of preparing 0.004 mol LSTCF fiber is as follows: stoichiometric amounts of La(NO_3_)_3_·6H_2_O (99.99%, Aladdin), Sr(NO_3_)_2_ (AR, Aladdin), Ti(OC_3_H_7_)_4_ (AR, Aladdin), Co(NO_3_)_2_·6H_2_O (99.99%, Aladdin), and Fe(NO_3_)_3_·9H_2_O (99.99%, Aladdin) were dissolved in 12 mL N, N-Dimethylformamide (DMF, ≥ 99.9%, Aladdin) solvent. Acetic acid (1 mL, AR, Aladdin) was added to reduce the pH value of the solution, inhibiting the hydrolysis of Ti(OC_3_H_7_)_4_. Then, 13 wt% (1.69 g) polyvinylpyrrolidone (PVP, M.W.≈130,000, Aladdin) was introduced into the solution to regulate the viscosity of the precursor and obtain the appropriate diameter of the fiber. The prepared solution was heated to 70 °C and continuously stirred for 24 h until it became transparent and viscous. Subsequently, the solution was transferred to a syringe to produce precursor fiber under the relative humidity of 40% and the environmental temperature of 25 °C, with a voltage of 15 kV. The rotating speed of the rotary drum was 500 r min^−1^, and the injecting rate of precursor was 3 μL min^−1^. The precursor fiber was kept at 400 °C in a Muffle furnace for 1 h (at a heating rate of 1 °C min^−1^) to remove organic matter and avoid fiber aggregation, followed by calcination at 900 °C for 3 h (at a heating rate of 5 °C min^−1^).

### Cell Fabrication

Electrolyte-supported single cells with a symmetrical configuration were used in this work. The substrate was commercial scandia-stabilized zirconia (SSZ, thickness ∼200 μm, SOFCMAN). A Gd_0.1_Ce_0.9_O_1.95_ (GDC, SOFCMAN) barrier layer was used to inhibit the reaction between the electrode and the electrolyte. GDC powder, ethyl cellulose, and terpineol were ball-milled in the ratio of 2 g: 0.08 g: 12 mL for 24 h to obtain a suspension, which was then uniformly spin-coated onto both sides of the SSZ electrolyte with a rotation speed of 1500 r min^−1^ for 50 s. The GDC layer was dried at 80 °C and then calcined at 1200 °C for 3 h. To improve thermal compatibility between the electrode and the electrolyte, GDC was added to the electrode. The LSTCF-GDC composite (LSTCF: GDC = 7: 3, weight ratio) was used as both the fuel and air electrodes. Terpineol and ethyl cellulose were mixed to make a viscous solution in a weight ratio of 95: 5. The LSTCF-GDC powder was ground with an appropriate amount of the above solution to prepare the electrode slurry. Finally, the electrode slurry was screen-printed onto both sides of the electrolyte, dried at 70 °C, and calcined at 800 °C for 3 h to obtain the single cells with a configuration of “LSTCF-GDC|GDC|SSZ|GDC|LSTCF-GDC”. The active area of the single cell was 0.196 cm^2^. Silver wires were fixed to electrodes by silver paste and used to collect current.

### Characterization

X-ray diffraction (XRD, Bruker D8 Advance A25, Germany) was conducted to analyze the crystal structure and the phase transition of the samples in the 2θ range of 20°–80°. The phase of the samples was preliminarily analyzed by Jade 6.0, and the structural information of the samples was confirmed by comparing them with the standard PDF card. The XRD data were refined by FullProf to obtain the crystal structure and cell parameters. X-ray photoelectron spectroscopy (XPS, Thermo Fisher ESCALAB Xi+ , USA) was performed to determine the different valences of Fe, Co, and O. Scanning electron microscope (SEM, Gemini500, Japan) was carried out to observe the microstructure of fibers, distribution of exsolved particles and cross-section of cells. The lattice and element distribution of the samples were tested by the Lorenz transmission electron microscope (Lorenz TEM, Talos-F200X, USA) at 200 kV with the point resolution of 0.23 nm and the lattice resolution of 0.12 nm. Lorenz TEM is equipped with an EDS spectrometer system to analyze the elemental composition of the sample surface and calculate the proportion of the elements. Automatic physical adsorption instrument (ASAP 2020 Plus HD88) was used to test the specific surface area of fibers by the Brunauer–Emmett–Teller (BET) method. CO_2_ temperature-programmed desorption (CO_2_-TPD) and H_2_ temperature-programmed reduction (H_2_-TPR) were carried out by Autochem II 2920 instrument (USA); the samples were LSTCF or La_0.6_Sr_1.2_Ti_0.2_Co_0.1_Fe_0.7_O_4−*δ*_ (R-LSTCF, reduced state) fiber with a mass of 150 mg. X-ray absorption fine structure (XAFS) data were obtained by the Shanghai synchrotron radiation facility (SSRF). Athena was used to calibrate and normalize the XAFS data, the R-space data of standard samples (Co-foil, Fe-foil, CoO, FeO, Co_2_O_3_, and Fe_2_O_3_), LSTCF and R-LSTCF were fitted by Artemis [33], and the wavelet transform of XAFS spectrum was obtained by MATLAB code developed by Manuel Muñoz and François Farges [34, 35].

### Electrochemical Measurements

Electrochemical performances of the LSTCF symmetrical cells were measured by an electrochemical workstation (Solartron Analytical SI 1260&1287, UK). Electrochemical impedance spectra (EIS) data were measured in the frequency range of 0.1 Hz to 1 MHz with an amplitude of 10 mV.

In the SOFC mode, the fuel electrode was first reduced in H_2_ (3% H_2_O) at 800 °C for 30 min with a flow rate of 30 mL min^−1^. The hybrid gas of H_2_ and CH_4_ was used as the fuel, and the air electrode was exposed to air with a flow rate of 30 mL min^−1^. The reversibility of the cell was measured by switching gases in the fuel electrode and the air electrode, using H_2_ (3% H_2_O) or 25% CH_4_-75% H_2_ (3% H_2_O) (30 mL min^−1^) as the fuel gas. Five cycles were carried out at ± 0.7 V for 110 h. Each cycle consists of two sections, each section lasting 10 h. After the initial 10 h operation, the two electrodes were fed with N_2_ (20 mL min^−1^) for 0.5 h. Then, fuel gas was supplied to the air electrode to convert it into a fuel electrode, and air was supplied to the fuel electrode to convert it into an air electrode for 0.5 h. After another 10 h operation, the air electrode and fuel electrode were switched back.

In the SOEC mode, the fuel electrode was treated differently depending on the reduction requirements for CO_2_ electrolysis: (i) In the first case, the fuel electrode was reduced in pure H_2_ (30 mL min^−1^) at 800 °C for 1 h in advance, followed by switching the fuel gas to pure CO_2_ (30 mL min^−1^). Simultaneously, the air electrode was exposed to dry air (30 mL min^−1^). (ii) In the second case, both the fuel electrode and air electrode were exposed to air (30 mL min^−1^) during the heating process. After reaching the target temperature, the fuel gas was switched to pure CO_2_ (30 mL min^−1^) for 1 h before the electrochemical test. For H_2_O electrolysis, the fuel electrode was reduced by the same method as in the SOFC mode. Then, the fuel gas was changed to 50% H_2_-50% H_2_O in a flow rates of 40 mL min^−1^, with air utilized as the oxidant (30 mL min^−1^).

### Computational Details

Density functional theory (DFT) simulations were implemented using the Vienna ab initio simulation package (VASP) software [36, 37]. In this study, the Perdew–Burke–Ernzerhof (PBE)-generalized gradient approximation (GGA) was used as the exchange–correlation functional [38]. The core electrons were described by projected augmented wave (PAW) pseudopotentials. During the calculation, a gamma point was exploited for all surface models, and the cut-off energy was installed at 400 eV. Spin-polarized calculations were performed to optimize all of the structures until the forces on each atom were within 0.05 eV Å^−1^, and the total energy convergence criterion of the self-consistent field method was set to be 10^–5^ eV. The six-layered SrFeO_3_ (SFO) (110) surface structure with one oxygen vacancy was modeled [39, 40], as shown in Fig. S1a. Similarly, the CoFe-nanoparticle-loaded reduced-Sr_2_FeO_4_ (CoFe@R-SFO) (103) was constructed to study the performance of the SFO after the nanoparticles exsolution (Fig. S1b). During the calculation, a vacuum thickness of 15 Å was used to minimize the interaction between the periodic slabs, and the three bottom atom layers were fixed to represent the bulk phase. The adsorption energies (Δ*E*_ads_) of CO_2_ molecules on the surface were calculated as follows [41]:

1 $$\begin{array}{*{20}c} {E_{{{\text{ads}}}} = E_{{{\text{surface}} + {\text{CO}}_{2} }} - E_{{{\text{surface}}}} - E_{{{\text{CO}}_{2} }} } \\ \end{array}$$

where ${E}_{\text{surface}+{\text{CO}}_{2}}$, ${E}_{\text{surface}}$ and ${E}_{{\text{CO}}_{2}}$ represent the energies for surface adsorbed with CO_2_, clean surface, and gas-phase CO_2_, respectively.

## Results and Discussion

### Structure and Reversible Phase Transition

**Fig. 1:**
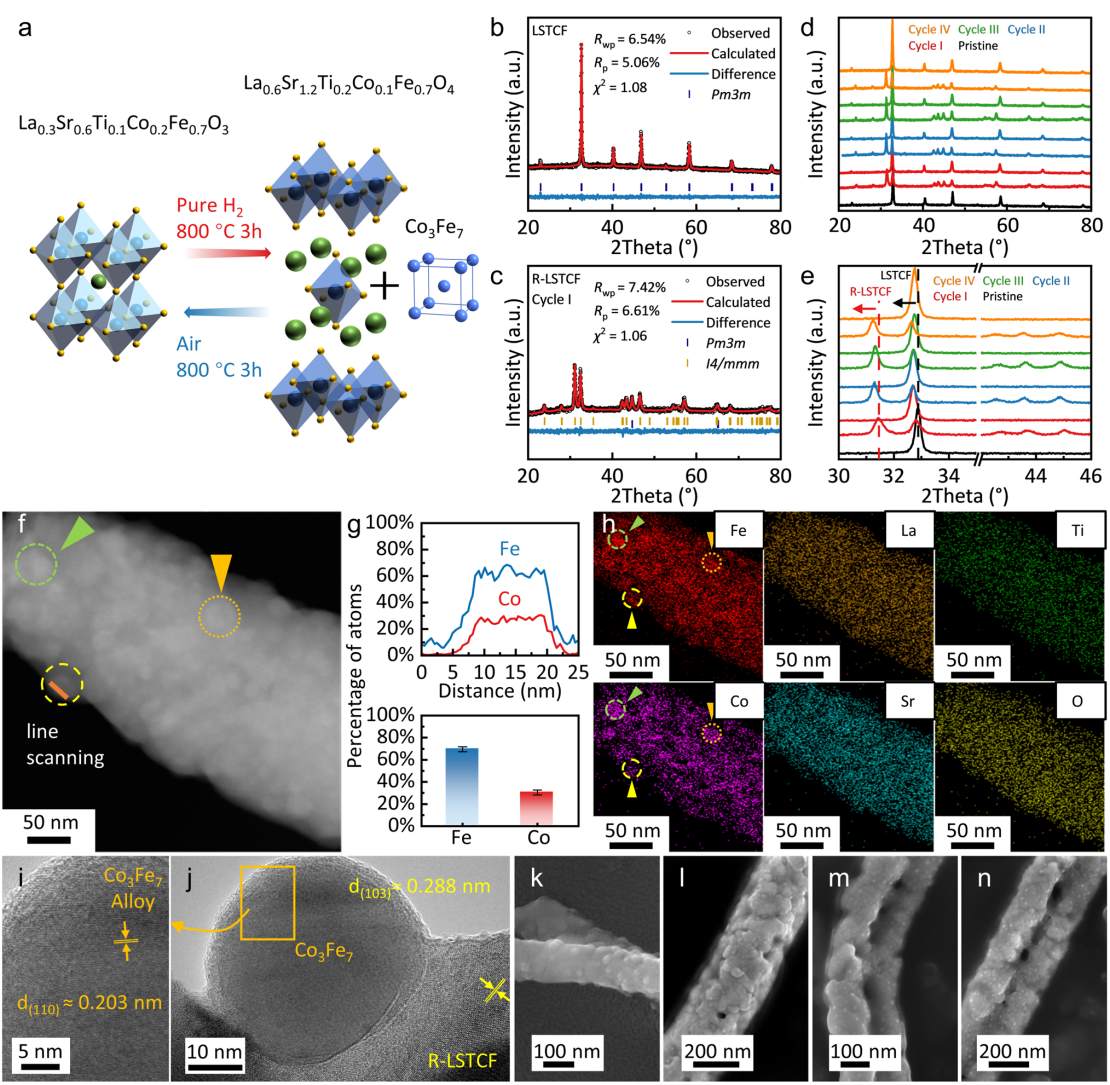
**a** Schematic illustration of the redox processes of LSTCF (A complete cycle includes both reduction and reoxidation: LSTCF was first reduced in pure H_2_ at 800 °C for 3 h to produce Cycle I R-LSTCF, Cycle I R-LSTCF was then reoxidized in air at 800 °C for 3 h, resulting in Cycle I LSTCF). XRD refinement patterns: **b** pristine LSTCF, **c** R-LSTCF after the first cycle. **d** XRD patterns of LSTCF during the redox cycles and **e** the enlarged view of the XRD patterns shown in Fig. 1d. Microstructure of the R-LSTCF fiber: **f** TEM image, **g** line scanning and atom content of the exsolved nanoparticles, **h** element distribution, **i** and **j** HR-TEM images of the exsolved Co_3_Fe_7_ nanoparticles. SEM images of the R-LSTCF with exsolved Co_3_Fe_7_ nanoparticles: **k** Cycle I R-LSTCF, **l** Cycle II R-LSTCF, **m** Cycle III R-LSTCF and **n** Cycle IV R-LSTCF

LSTCF showed high structure reversibility under oxidizing and reducing atmospheres. Four redox cycles were carried out to investigate the reversible phase transition of LSTCF. The single redox cycle treatment process is illustrated in Fig. 1a: the synthesized LSTCF was first reduced in pure H_2_ at 800 °C for 3 h and then oxidized in air at 800 °C for 3 h. After reduction, LSTCF transformed from ABO_3_ perovskite to A_2_BO_4_ perovskite with exsolved Co_3_Fe_7_ alloy nanoparticles (Figs. 1b, c and S2). While during the subsequent oxidation process, the Co_3_Fe_7_ alloy was oxidized and re-dissolved into the matrix, regenerating the ABO_3_ perovskite. XRD patterns of the pristine LSTCF and the LSTCF after each oxidation or reduction treatment are shown in Fig. 1d, e. It is found that LSTCF underwent a phase transition following the order ABO_3_ → A_2_BO_4_ + Co_3_Fe_7_ → ABO_3_ → A_2_BO_4_ + Co_3_Fe_7_ without any other impurity phases, demonstrating the reversible phase transition of LSTCF*.* Similar phenomenon has been observed in Sr_2_Fe_1.5_Mo_0.5_O_6−*δ*_ (SFM)-based double perovskites [23, 42]. Detailed structure information of LSTCF (oxidized state) and R-LSTCF (reduced state) after each oxidation or reduction treatment is shown in Fig. S3 and Tables S1, S2. LSTCF exhibits a single ABO_3_ cubic perovskite phase with a space group of *Pm3m*, while the R-LSTCF consists of a primary A_2_BO_4_ perovskite phase with a space group of *I4/mmm* and a secondary Co_3_Fe_7_ phase with a space group of *Pm3m* [43]. The reversible phase transition process of LSTCF is:

2 $$\begin{array}{*{20}c} {{\text{2 La}}_{{{0}{\text{.3}}}} {\text{Sr}}_{{{0}{\text{.6}}}} {\text{Ti}}_{{{0}{\text{.1}}}} {\text{Co}}_{{{0}{\text{.2}}}} {\text{Fe}}_{{{0}{\text{.7}}}} {\text{O}}_{3} \rightleftarrows {\text{La}}_{{{0}{\text{.6}}}} {\text{Sr}}_{1.2} {\text{Ti}}_{{{0}{\text{.2}}}} {\text{Co}}_{{{0}{\text{.1}}}} {\text{Fe}}_{{{0}{\text{.7}}}} {\text{O}}_{4} { + 0}{\text{.1 Co}}_{3} {\text{Fe}}_{7} + {\text{O}}_{2} } \\ \end{array}$$

Both ABO_3_ and A_2_BO_4_ perovskites contain A-site defects. The stoichiometric ratios of A-site to B-site in the perovskites are 0.9 and 1.8 for LSTCF and R-LSTCF, respectively. The A-site defect promotes the exsolution of B-site metal ions and inhibits the enrichment of the AO phase by reducing the nucleation barrier and introducing oxygen vacancy [44]. It is worth noting that after redox cycling, the XRD characteristic peaks of R-LSTCF slightly shift to lower angles (Fig. 1e), which may be attributed to the loss of lattice oxygen [45].

The reduction process of different metal ions of LSTCF was investigated by H_2_-TPR. In Fig. S4, TPR results of LSTCF show four obvious characteristic peaks, representing Co^3+^ → Co^2+^, Fe^3+^ → Fe^2+^, Ti^4+^ → Ti^3+^ and the Co_3_Fe_7_ alloy exsolution (Co^2+^ → Co^0^ and Fe^2+^ → Fe^0^), respectively, from the low temperature to high temperature [46, 47].

Figure S5 shows the SEM images of LSTCF and R-LSTCF fibers during the redox cycling. The pristine LSTCF fibers showed a network form with a smooth surface, and no obvious pores or depressions were observed (Fig. S5a). After the first reduction, exsolved spherical nanoparticles were uniformly distributed on the surface of the R-LSTCF fibers, which appeared smooth and without obvious pores (Fig. S5b). After the subsequent redox cycling, the fiber surface became coarse with uneven pores. As shown in Fig. S6, after cycling, the average diameter of LSTCF fibers increased slightly from 183.4 nm (Pristine) to 186.5 nm (Cycle IV), and the average diameter of R-LSTCF increased from 179.6 nm (Cycle I) to 181.4 nm (Cycle IV). Figure S7 presents the specific surface area of fibers by BET method. After four redox cycles, the specific surface areas of LSTCF and R-LSTCF fibers increased from 5.49 to 7.18 m^2^ g^−1^ and from 5.63 to 7.54 m^2^ g^−1^, respectively.

### In Situ Exsolution and Re-Dissolution

As shown in Fig. 1f, numerous nanoparticles with diameters of ~ 15‒30 nm were exsolved from the R-LSTCF fiber after reduction. Line scanning of the nanoparticle determined the composition of Co and Fe with a ratio of ~ 3:7 (the Co content is 30.44% ± 2.19%, and the Fe content is 69.56% ± 2.19%) (Fig. 1g). The uneven distribution of Co and Fe on the surface of R-LSTCF fibers and the uniform distribution of La, Sr, Ti, and O are shown in Fig. 1h. High-resolution transmission electron microscope (HR-TEM) images of the Co_3_Fe_7_@R-LSTCF heterostructure are shown in Fig. 1i, j. The lattice spacing of the fiber matrix is about 0.288 nm, corresponding to the (103) plane of the R-LSTCF A_2_BO_4_ perovskite [48], and the lattice spacing of the nanoparticle is about 0.203 nm, which corresponds to the (110) plane of the Co_3_Fe_7_ crystal [43].

The morphology of Co_3_Fe_7_ nanoparticles and their diameter distribution are shown in Figs. 1k–n and S8, respectively. The average diameter of the exsolved alloy nanoparticles is: 27.5 nm (Cycle I) > 20.7 nm (Cycle II) > 19.0 nm (Cycle III) > 18.6 nm (Cycle IV). The distribution density of nanoparticles is: 53.1 μm^−2^ (Cycle I) < 153.3 μm^−2^ (Cycle II) < 182.8 μm^−2^ (Cycle III) < 205.6 μm^−2^ (Cycle IV). Obviously, the average diameter of nanoparticles exhibits a negative correlation with the surface distribution density. After multiple redox cycles, the fiber surface became rougher and more defects appeared, leading to an increase in the number of nucleation sites for the exsolution of nanoparticles. The exsolution may be dominated by the nucleation process rather than the growth process [49, 50]. The exsolved Co_3_Fe_7_ nanoparticles with increased distribution density and reduced particle size can benefit the catalytic activity and stability of the electrode material.

**Fig. 2:**
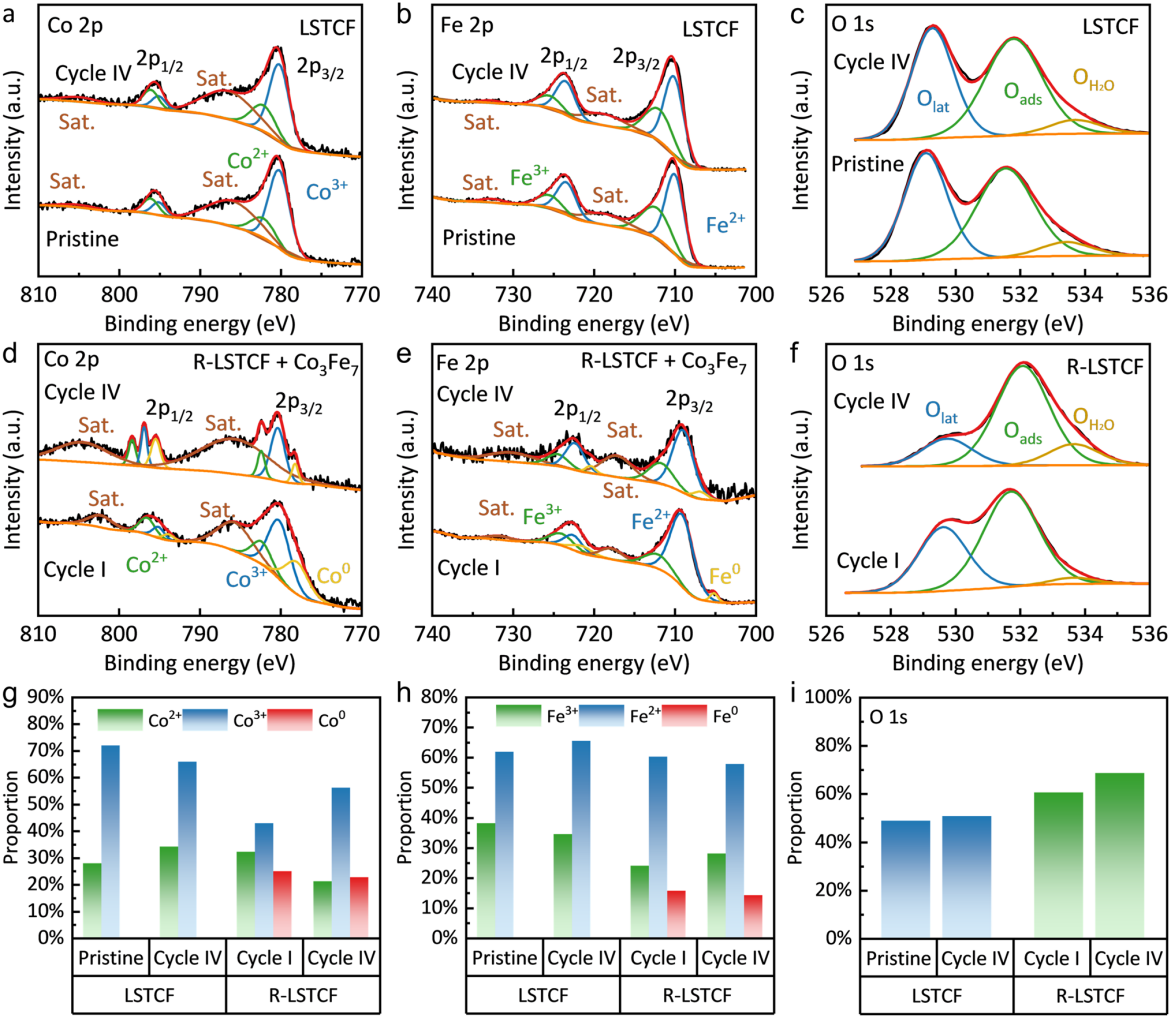
XPS spectra of **a** LSTCF Co 2*p*, **b** LSTCF Fe 2*p*, **c** LSTCF O 1*s*, **d** R-LSTCF Co 2*p*, **e** R-LSTCF Fe 2*p* and **f** R-LSTCF O 1*s*. The valence distribution of **g** Co element, **h** Fe element and **i** differences in the proportion of O_ads_

The exsolution of Co_3_Fe_7_ nanoparticles and the surface chemistry of the electrode material were further characterized by XPS. The XPS spectra of Co in LSTCF and R-LSTCF are shown in Fig. 2a, d, respectively. For LSTCF, the element Co exists in the form of Co^2+^ and Co^3+^, and for R-LSTCF, additional characteristic peaks located near the binding energies of 777.8 and 793.6 eV, corresponding to Co^0^ 2*p*_3/2_ and Co^0^ 2*p*_1/2_, respectively, are observed [43, 51]. Similarly, Fe^0^ (Fe^0^ 2*p*_3/2_ and Fe^0^ 2*p*_1/2_ with the binding energies of 706.1 and 720.6 eV, respectively) is found in R-LSTCF in addition to Fe^2+^ and Fe^3+^ (Fig. 2b, e) [43, 52]. As shown in Fig. 2c, f, the characteristic peaks of O 1*s* contain three major peaks at 529.2, 531.7, and 533.5 eV, corresponding to lattice oxygen (O_lat_), adsorbed oxygen (O_ads_), and weakly adsorbed oxygen (O_H2O_), respectively [29]. Compared to LSTCF, the content of Co^3+^ and Fe^3+^ in R-LSTCF decreases (Fig. 2g, h). The high-valence ions were reduced to the corresponding low-valence ions, and the low-valence ions were reduced to form nanoparticles on the fiber surface. At the same time, the O_lat_ in the BO_6_ octahedron reacted with H_2_, leading to an increase in the proportion of O_ads_ and the concentration of oxygen vacancies (Fig. 2i), which is beneficial to the oxygen-related electrochemical process in the fiber electrode.

**Fig. 3:**
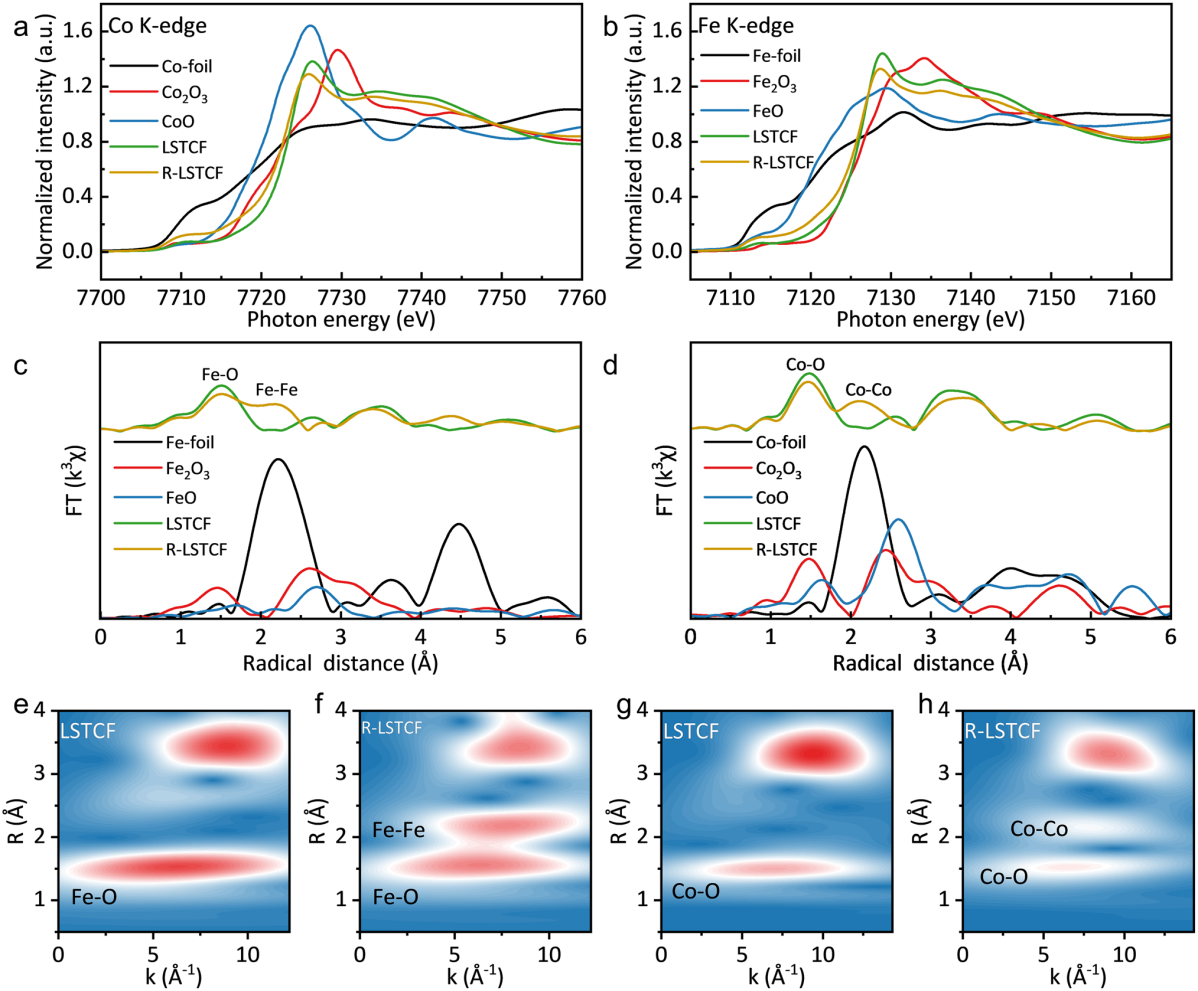
**a** Co and **b** Fe K-edge X-ray absorption near edge structure (XANES) spectra. **c**, **d** Fourier transforms of k^3^-weighted EXAFS spectra. **e**, **h** Wavelet transforms of k^3^-weighted EXAFS spectra: **e** has the same contour color standard as **f**, **g** has the same contour color standard as **h**

The effect of reversible phase transition between LSTCF and R-LSTCF on the valence and coordination number of Co and Fe was studied by XAFS. It can be seen that R-LSTCF shows obvious pre-edge peaks at ~ 7710 eV (Fig. 3a) and ~ 7113 eV (Fig. 3b) compared with LSTCF, indicating the decrease in molecular symmetry of the perovskite matrix changed from a cubic structure (ABO_3_) to tetragonal structure (A_2_BO_4_). In addition, the valence of Co and Fe decreases as the absorption edge of R-LSTCF shows a redshift compared to LSTCF, which is consistent with the XPS analysis shown above [47, 53]. The Co K-edge energy decreased to 7723.9 eV from 7724.4 eV and the Fe K-edge energy decreased to 7126.5 eV from 7126.8 eV, accompanied by a decrease intensity of the white line due to less well-defined energy levels in R-LSTCF [54]. Extended X-ray absorption fine spectra (EXAFS) at Co and Fe K-edge were used to confirm the exsolution of the alloy and the change of B-site elements coordination, as shown in Fig. 3c, d. The R-LSTCF shows peaks representing Co–Co and Fe–Fe bonds between 2 and 3 Å, corresponding to the specific peaks of Co-foil and Fe-foil with a bond length of 2.50 Å (Figs. S9 and S10, Tables S3 and S4). The peaks located in 1 ~ 2 Å in R-space correspond to Co–O and Fe–O bonds, and the bond lengths were determined to be 1.91 and ~ 2.00 Å, respectively, by shell fitting and the comparison with standard samples. The peak strength of Co–O bond and Fe–O bond of R-LSTCF is decreased compared to LSTCF, indicating a decrease in the metal coordination number at the B-site. The fitting results show that after reduction, the coordination number of Co–O bond decreased from 5.5 to 4.4, and the coordination number of Fe–O bond decreased from 5.9 to 5.5. Unsaturated coordination indicates an increase in oxygen vacancy concentration [55], which is consistent with the XPS results. Wavelet-transformed EXAFS also shows the presence of the alloy structure and the change in the coordination number of the B-site elements of the perovskite (Fig. 3e–h).

### Electrochemical Performance

**Fig. 4:**
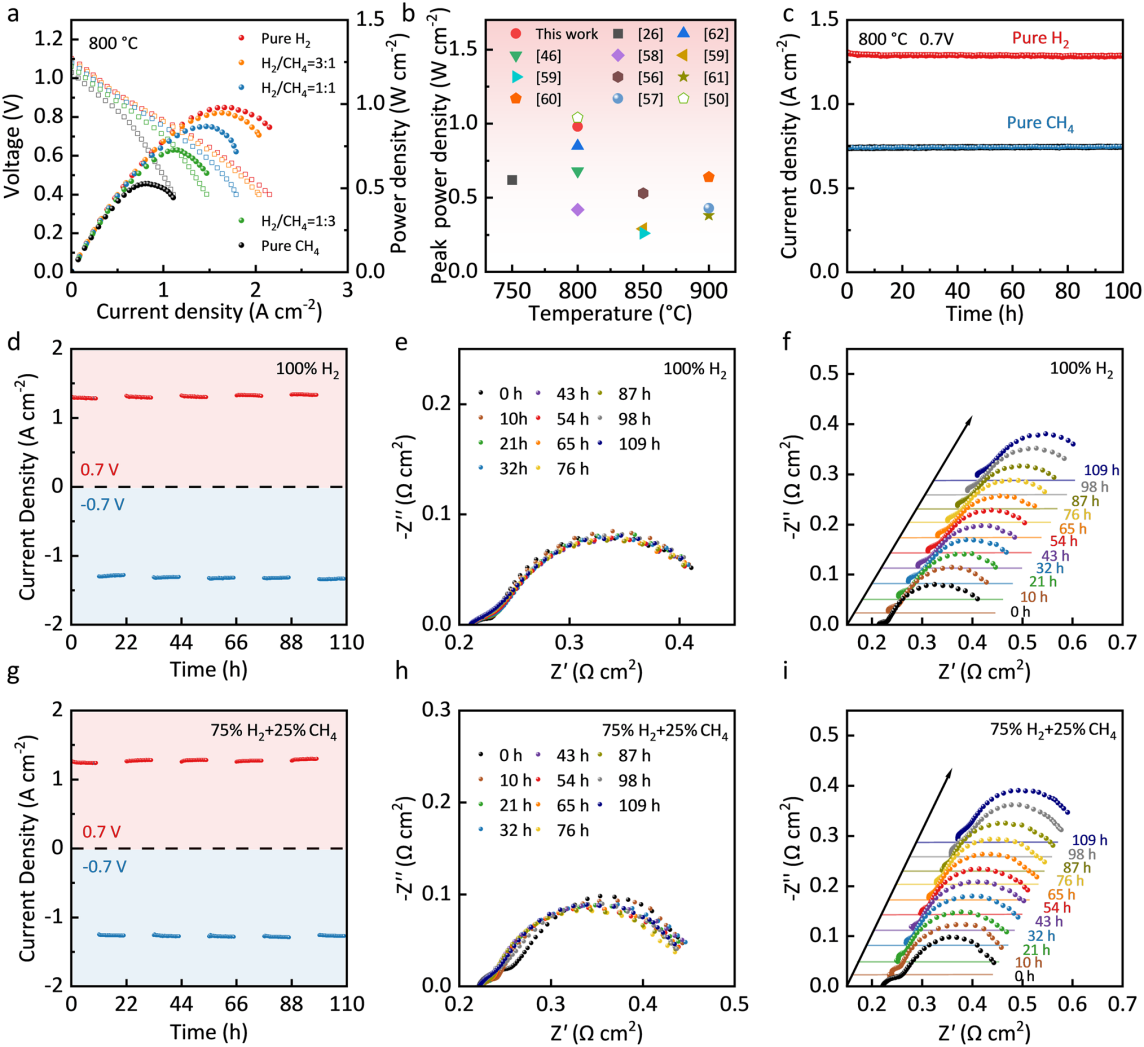
**a** *I*–*V*–*P* curves of the single cell at 800 °C in the fuel cell mode using H_2_-CH_4_ (3% H_2_O) as the fuel and air as the oxidant. **b** Peak power density comparison of state-of-the-art SSZ or YSZ electrolyte-supported SOFCs [26, 46, 50, 56–62]. **c** Stability of the cell using H_2_ and CH_4_ as the fuel at 800 °C and 0.7 V. Reversible operation of the single cell by switching the fuel and air electrodes with pure H_2_ (3% H_2_O) as the fuel at 800 °C and ± 0.7 V (the cell electrodes were switched after every 10 h): **d** the cell voltage as a function of time, **e**, **f** Nyquist curves of the EIS. Reversible operation of the single cell with 75% H_2_-25% CH_4_ (3% H_2_O) as the fuel at 800 °C and ± 0.7 V (the cell electrodes were switched after every 10 h): **g** the cell voltage as a function of time, **h**, **i** Nyquist curves of the EIS

The electrochemical performance and stability of the LSTCF-GDC electrode were first evaluated in the SOFC mode using SSZ electrolyte-based cells with a symmetrical configuration of “LSTCF-GDC|GDC|SSZ|GDC|LSTCF-GDC”. The air electrode was exposed to air with a flow rate of 30 mL min^−1^, and the fuel electrode was fed by the H_2_-CH_4_ hybrid gas (3% H_2_O) at a constant flow rate of 30 mL min^−1^, with H_2_ contents of 0%, 25%, 50%, 75%, and 100%, respectively. As shown in Fig. 4a, the H_2_ content has a positive correlation with the cell performance. As increasing the H_2_ content from 0 to 100%, the peak power density (*P*_Max_) of the cell increased from 0.53 to 0.98 W cm^−2^ at 800 °C. Figure S11 shows the constant Ohmic resistance and decreased polarization resistance (*R*_p_) of the cell as increasing the H_2_ content, which is consistent with the cell performance as shown in Fig. 4a. The single cell with the LSTCF-GDC symmetrical electrode shows higher performance than most of the reported SSZ or yttrium-stabilized zirconia (YSZ) electrolyte-supported cells in the SOFC mode (Fig. 4b) [26, 46, 50, 56–62]. Details of the comparison are listed in Table S5. Figure 4c shows the time dependence of the current densities of cells operated under H_2_ and CH_4_, with no obvious fluctuation or decay of current densities observed for 100 h, demonstrating the high stability of the LSTCF-GDC in both oxidizing and reducing atmospheres, especially the excellent carbon resistance.

The reversibility of the LSTCF-GDC electrodes was first studied by switching the gas in each electrode, i.e., switching the fuel electrode and the air electrode. As shown in Fig. S12, after 10 h of operation, the gases in both electrodes were shut off and purged with N_2_ for 0.5 h, and then, the fuel electrode was switched to an air electrode by feeding air while the air electrode was switched to a fuel electrode by feeding fuel for the next 10 h operation. Under realistic operational conditions, the cell degradation caused by sulfur poisoning or coking of the fuel electrode can be mitigated by operating the fuel electrode as an air electrode, allowing air to remove the contaminants. The flow rates of fuel gas and air were 30 mL min^−1^. As shown in Fig. 4d, g, no degradation in the cell performance is observed either using pure H_2_ or 75%H_2_-25%CH_4_ as the fuel, confirming the high reversibility of the LSTCF-GDC electrode. It is worth noting that compared with the first cycle, the cell performance showed a slight increase trend from the second cycle, with the current density increased and the *R*_p_ reduced (Fig. 4d–i). The improved cell performance may be due to the increased distribution density and reduced particle size of the exsolved Co_3_Fe_7_ nanoparticles, which boosted the reactive area for ORR and OER. Furthermore, the improved oxygen vacancy concentration of the LSTCF-GDC electrodes after the redox cycling may contribute to the performance improvement. Compared to previous electrode materials with reversible phase transitions, the most notable advantage of LSTCF-GDC is the refinement and density development of the nanoparticles by surface reconstruction [45].

**Fig. 5:**
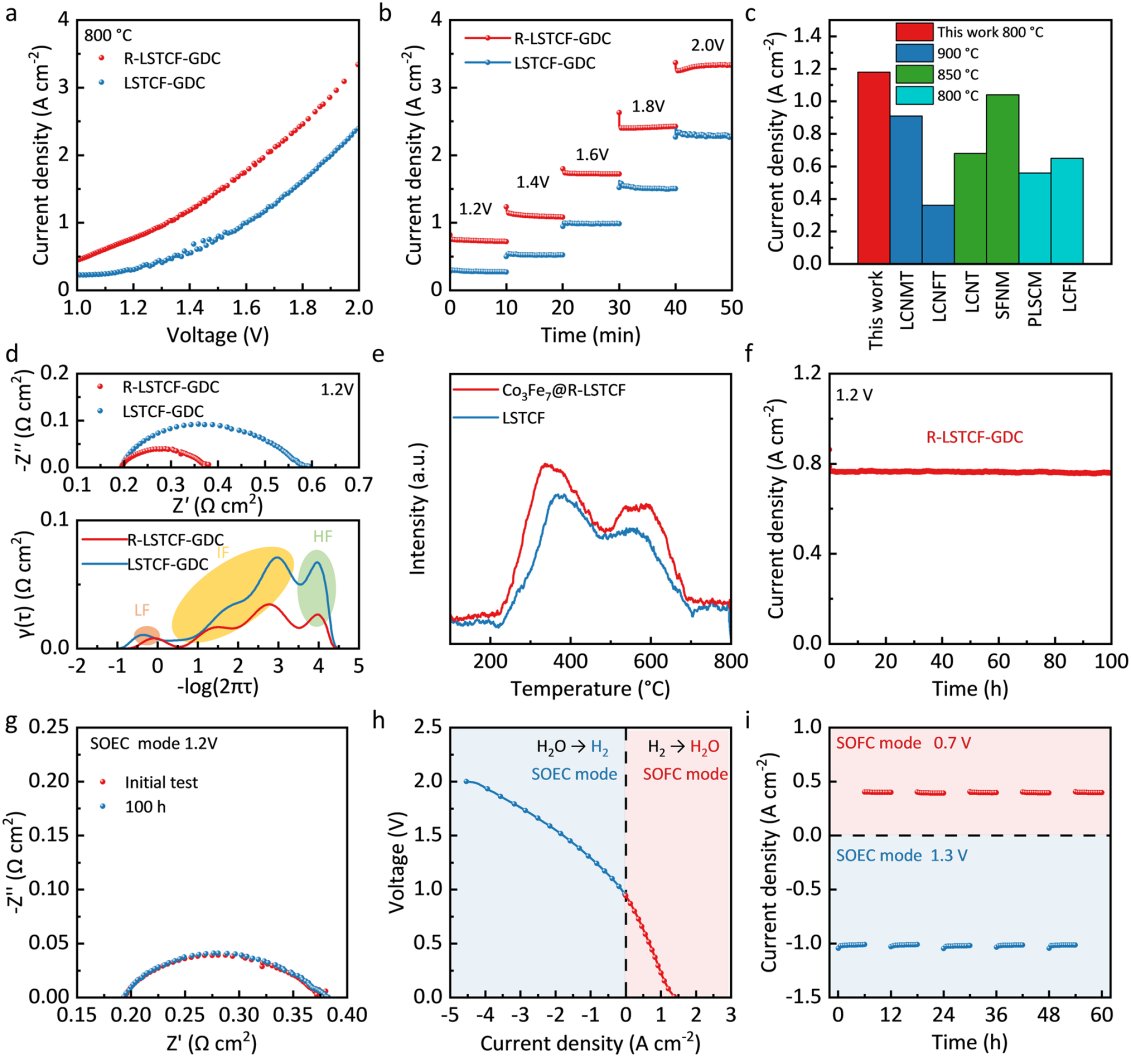
Electrochemical performance of LSTCF-GDC and R-LSTCF-GDC cells for CO_2_ electrolysis (100% CO_2_, 30 mL min^−1^) at 800 °C: **a** *I*–*V* curves, **b** potentiostatic tests at 1.2–2 V, **c** current density comparison of state-of-the-art SSZ or YSZ electrolyte-supported SOECs for CO_2_ electrolysis at 1.4 V [16, 22, 57, 59, 63, 64], **d** Nyquist curves of the EIS and DRT plots measured at 1.2 V, **e** CO_2_-TPD results of LSTCF and R-LSTCF fibers, **f** stability of the R-LSTCF-GDC cell at 1.2 V, **g** EIS of the R-LSTCF-GDC cell before and after the stability test measured at 1.2 V. Electrochemical performance of the single cell in the RSOC mode for H_2_O electrolysis with 50% H_2_-50% H_2_O in the fuel electrode at 800 °C: **h** *I*–*V* curve, **i** stability in the reversible mode at 0.7 and 1.3 V (the operation mode was changed after every 6 h)

The reversibility and performance of the LSTCF-GDC electrodes were further studied by testing the single cells in the electrolysis mode for CO_2_ and H_2_O electrolysis. The air electrode was exposed to air, and the fuel electrode was fed by pure CO_2_ at a constant flow rate of 30 mL min^−1^. Depending on whether the fuel electrode was reduced or not before the electrochemical measurements, the individual cells are referred to as LSTCF-GDC or R-LSTCF-GDC cells. The R-LSTCF-GDC cell shows much higher performance than that of the LSTCF-GDC cell. For example, at 800 °C and an applied voltage of 1.2 V, the current density of the R-LSTCF-GDC cell was 0.77 A cm^−2^ (Fig. 5a), which is more than twice of the LSTCF-GDC cell (0.30 A cm^−2^). Figure 5b shows the potentiostatic measurements of the cells for CO_2_ electrolysis. The current densities of the R-LSTCF-GDC cell were 0.72, 1.09, 1.72, 2.40, and 3.33 A cm^−2^ at voltages of 1.2, 1.4, 1.6, 1.8, and 2.0 V, respectively, much higher than those of the LSTCF-GDC cell. The single cell with the R-LSTCF-GDC fuel electrode shows higher performance than most of the SSZ or YSZ electrolyte-supported cells for direct CO_2_ electrolysis (Fig. 5c) [16, 22, 57, 59, 63, 64]. Details of the comparison are listed in Table S6. The LSTCF-GDC electrode outperforms conventional powder LST-based electrodes may due to its high porosity, abundant active sites, and accelerated ion and electron conductivity [65]. The well-distributed nanoparticles created by the surface reconstruction of the fibers also make the cell with the LSTCF-GDC electrode has obvious benefits in peak power density, electrolytic current, and stability when compared to the cell with the LST-based fiber electrode [61].

Distribution of relaxation time (DRT) analyses of the EIS was used to obtain a better understanding of the electrode reactions for CO_2_ electrolysis [66]. EIS and the corresponding DRT plots of the LSTCF-GDC and R-LSTCF-GDC cells are shown in Fig. 5d. The DRT plot is composed of distinct peaks observed in high frequency (HF), intermediate frequency (IF), and low frequency (LF). In general, the HF peak is associated with the charge transfer processes across electrolyte–electrode interfaces; the IF peak is related to the surface oxygen exchange processes (e.g., oxygen/carbon dioxide adsorption, dissociation, and surface transport); and the LF peak is attributed to the mass transfer processes in the electrode [16, 21]. As shown, the resistance in the IF dominates the total polarization resistance of the LSTCF-GDC and R-LSTCF-GDC cells. Compared with the LSTCF-GDC cell, the R-LSTCF-GDC cell shows significantly reduced resistance in IF, indicating the accelerated surface exchange process (e.g., CO_2_ adsorption and/or dissociation) [16]. Figure 5e shows the CO_2_-TPD curves of LSTCF and R-LSTCF fibers. The peaks in the low temperature (200–400 °C) and high temperature (500–700 °C) regions correspond to the physical adsorption and chemical adsorption processes of CO_2_, respectively [22, 67]. R-LSTCF shows higher CO_2_ adsorption capability than LSTCF (Fig. 5e), which is consistent with the DRT analysis.

The R-LSTCF-GDC cell shows a high faradaic efficiency of 97.3% and a high CO yield of 5.22 mL cm^−2^ min^−1^, higher than those of the LSTCF-GDC cell (95.2% and 2.01 mL cm^−2^ min^−1^, Fig. S13). The current density of the R-LSTCF-GDC cell shows no obvious decrease in the 100-h test at 1.2 V (Fig. 5f), demonstrating excellent stability of the R-LSTCF-GDC for CO_2_ electrolysis, which is also confirmed by the identical EIS and *I*–*V* curves measured before and after the stability test (Figs. 5g and S14). The cross-section SEM image of the R-LSTCF-GDC cell after the stability test shows a dense electrolyte, a fibrous R-LSTCF-GDC electrode, and good adhesion between the electrode and the electrolyte layer (Fig. S15).

The R-LSTCF-GDC cell also shows high performance and stability in the reversible mode for power generation and H_2_O electrolysis, achieving a high current density of − 1.03 A cm^−2^ at 1.3 V and 800 °C while maintaining excellent durability for over 60 h without variation in EIS and microstructure (Figs. 5h, i and S16, S17).

**Fig. 6:**
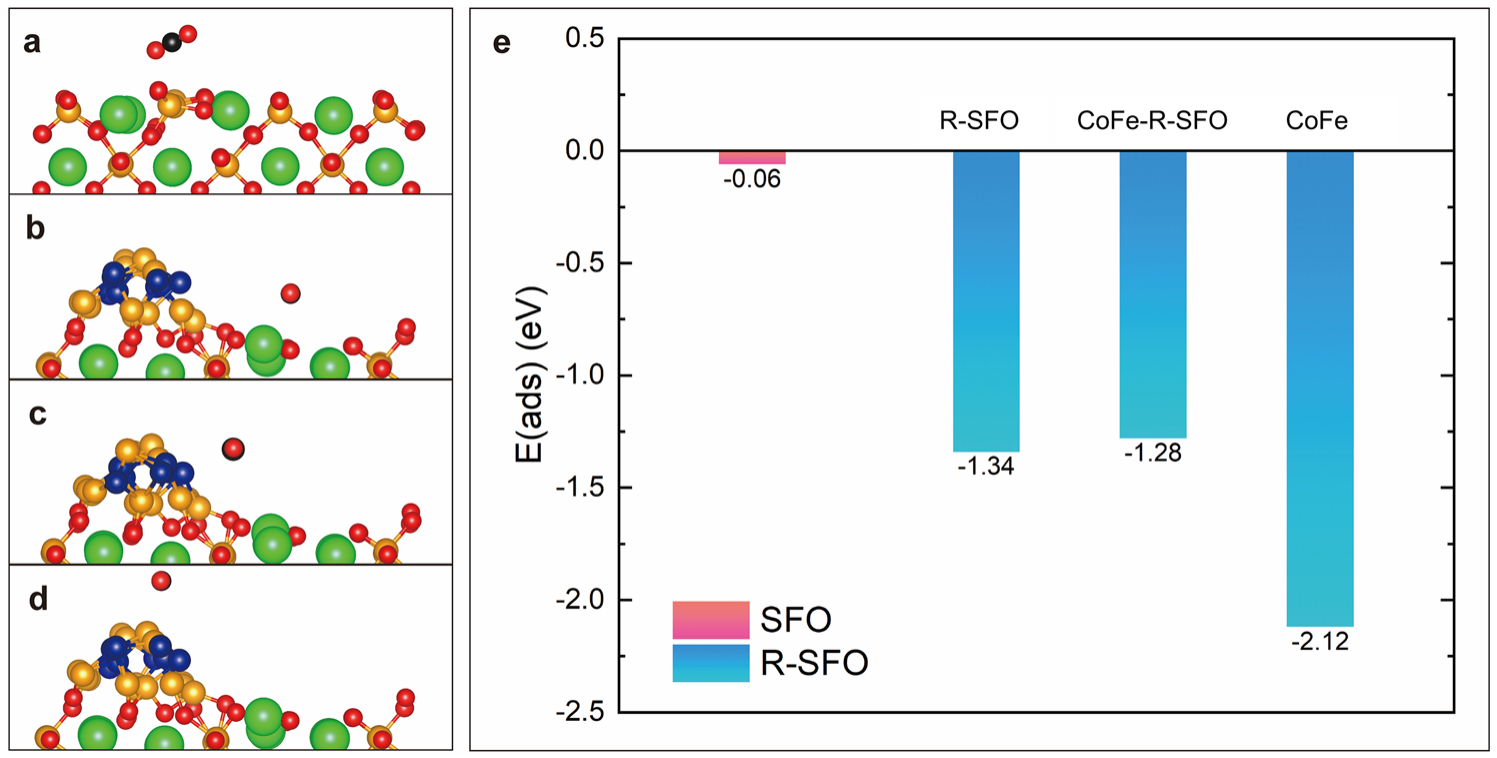
Schematic illustration of the CO_2_ adsorption process at **a** SFO (110) surface, **b** R-SFO (103) surface, **c** CoFe‒R-SFO interface, and **d** CoFe nanoparticles. **e** Comparison of CO_2_ adsorption energies at different sites shown in **a**–**d**. In the crystal structure, atoms are color-coded as follows: oxygen (red), strontium (green), iron (brown), carbon (black) and cobalt (blue). (Color figure online)

Density functional theory calculations were carried out to understand the high activity of LSTCF for CO_2_ electrolysis. Based on the XRD and TEM characterization results, surface models of SFO (110) and RP-Sr_2_FeO_4_ (R-SFO, 103) with CoFe nanoparticles (CoFe@R-SFO) were constructed. Two (110) surfaces of CoFe nanoparticles were exposed as the active surfaces for CO_2_ adsorption. Details of the modeling and calculation method are shown in computational details (Sect. 2.5)and Fig. S1. Figure 6a–d shows the CO_2_ adsorption processes on the surface models. As shown in Fig. 6e, the CO_2_ adsorption energy (*E*_ads_) on the CoFe@R-SFO surface (including the R-SFO surface, CoFe–R-SFO interface, and CoFe surface) is extremely lower than that on SFO (i.e., − 1.28 to − 2.12 eV on CoFe@R-SFO v.s. − 0.06 eV on SFO), indicating the much higher CO_2_ adsorption capability of CoFe@R-SFO. This result is consistent with the experimental results as shown above. Meanwhile, the CO_2_ adsorption on CoFe is more favorable than that on the CoFe‒R-SFO interface and the R-SFO substrate. The exsolved CoFe nanoparticles enhance the catalytic activity for CO_2_ electrolysis through promoting the CO_2_ adsorption process [23, 68].

## Conclusions

In summary, we have developed a symmetrical LSTCF fiber electrode with remarkable electrocatalytic activity and durability. The surface morphology and structure of the LSTCF fiber and the distribution of exsolved Co_3_Fe_7_ nanoparticles were reconstructed by a reversible phase transition process. The exsolution of Co_3_Fe_7_ nanoparticles increased the concentration of oxygen vacancies on the surface of the perovskite matrix. The experimental and computational results confirmed the high CO_2_ adsorption capacity of the Co_3_Fe_7_@R-LSTCF electrode, which may contribute to the high performance of RSOCs for CO_2_ electrolysis. This study demonstrated the potential application of LSTCF as an efficient and durable electrode material for energy conversion and storage.

## Supplementary Information

Below is the link to the electronic supplementary material.

Supplementary file1 (DOCX 13512 KB)
