# Supplementary material for: Tailoring the Reversible Phase Transition of Perovskite Nanofiber Electrodes for High-Performance and Durable Reversible Solid Oxide Cells
Source: Nanomicro Lett. 2025 Feb 17;17:150. doi: 10.1007/s40820-024-01600-4 (PMC11832975; doi:10.1007/s40820-024-01600-4)
Supplement: Supplementary file 1 — Supplementary file1 (DOCX 13512 KB) [file 40820_2024_1600_MOESM1_ESM.docx]

Supporting Information for

**Tailoring the Reversible Phase Transition of Perovskite Nanofiber Electrodes for High-Performance and Durable Reversible Solid Oxide Cells**

Chaofan Yin^1, 2^, Jiaming Yang^1, 3^, Jiangyuan Feng^2^, Yueyue Sun^1^, Zhengrong Liu^1^, Junkai Wang^1^, Jiajia Cui^1, 4^, Zixuan Xue^1, 2^, Liang Zhang^2, 5^, Yucun Zhou^2,^ *, Jun Zhou^1,^ *, Liangfei Xu^2, 5^, Kai Wu^1^ and Jianqiu Li^2, 5^

^1^ Center of Nanomaterials for Renewable Energy, State key laboratory of electrical insulation and power equipment, Xi’an Jiaotong University, Xi’an, Shaanxi 710049, P. R. China

^2^ Beijing Huairou Laboratory, Beijing 101400, P. R. China

^3^ Xi'an Thermal Power Research Institute Co., Ltd, Xi’an, Shaanxi 710054, P. R. China

^4^ School of Materials Science and Engineering, Xi’an University of Technology, Xi'an, Shaanxi 710048, P. R. China

^5^ School of Vehicle and Mobility, Tsinghua University, Beijing 100084, P. R. China

*Corresponding authors. E-mail: [zhouyucun@hrl.ac.cn](mailto:zhouyucun@hrl.ac.cn) (Yucun Zhou); [zhoujun@mail.xjtu.edu.cn](mailto:zhoujun@mail.xjtu.edu.cn) (Jun Zhou)

**Supplementary Figures and Tables**


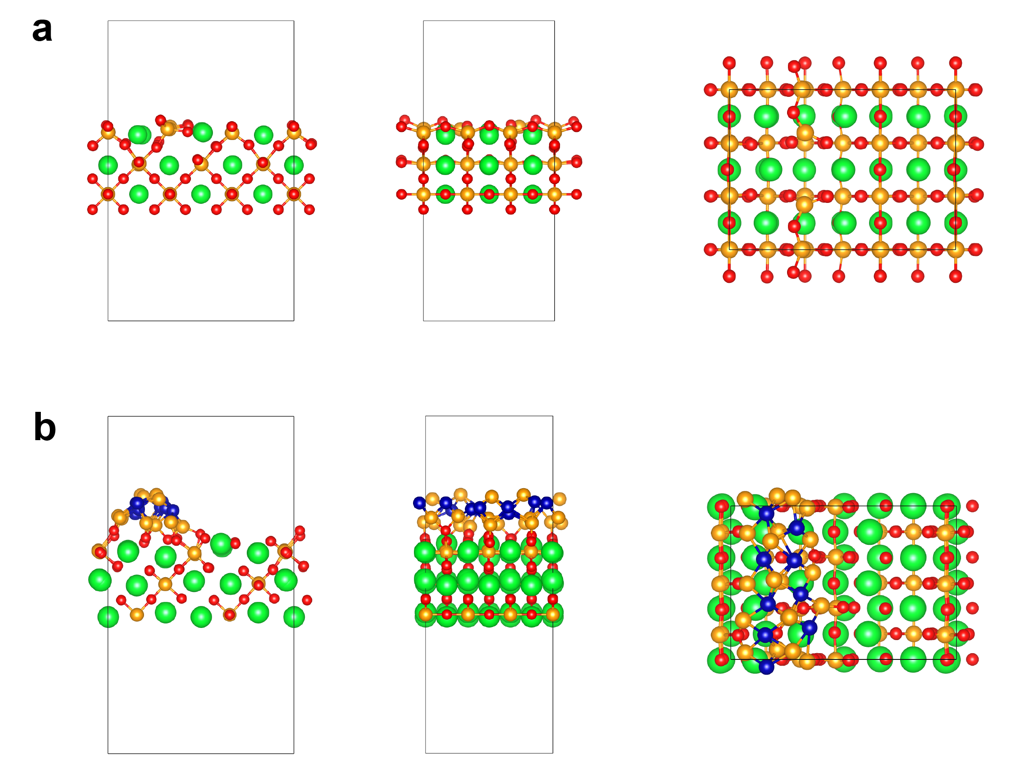


**Fig. S1** Surface structures of **a** SFO (110) and **b** CoFe@R-SFO (103) from side and top viewpoints, respectively


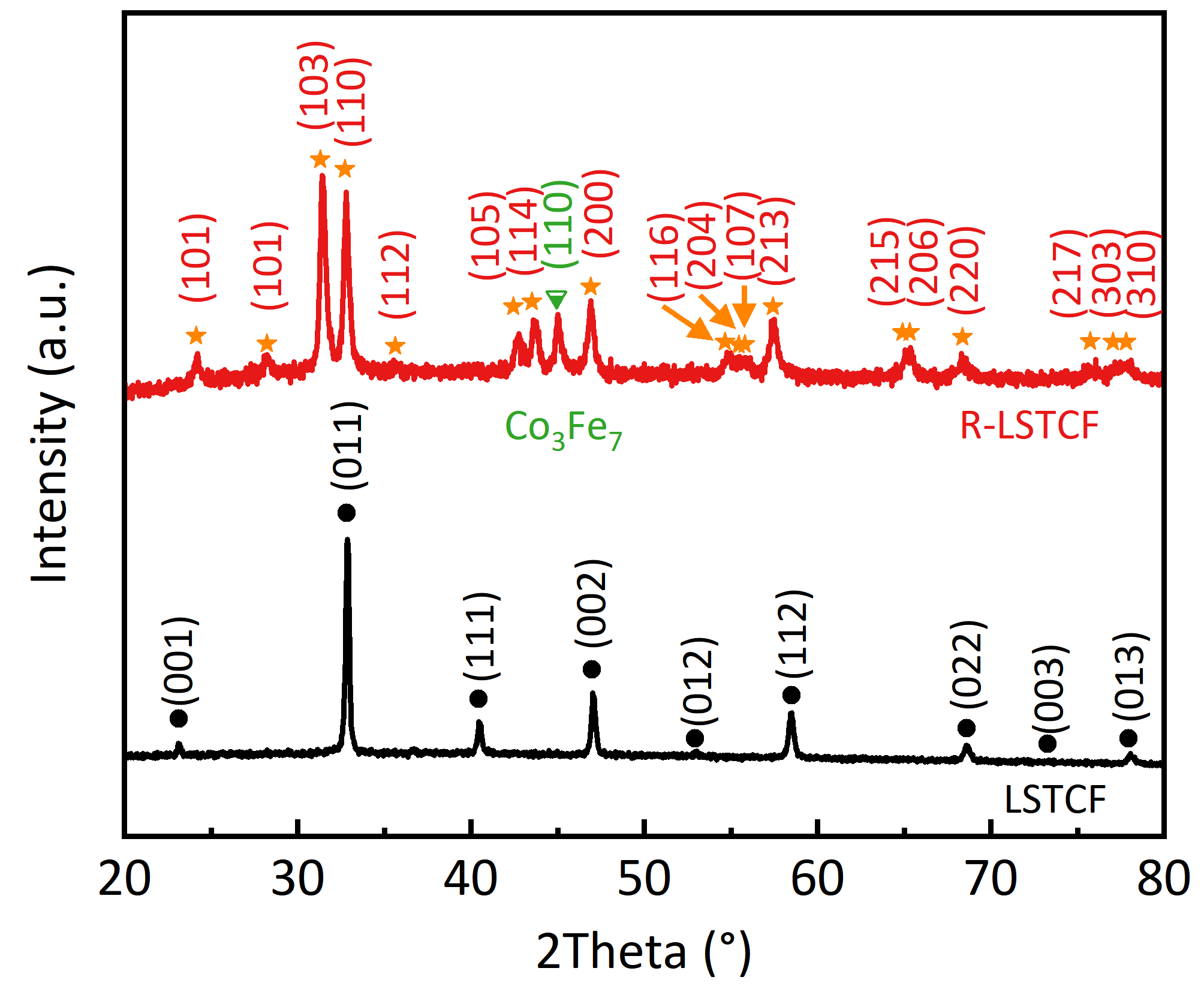


**Fig. S2** XRD patterns of LSTCF and R-LSTCF


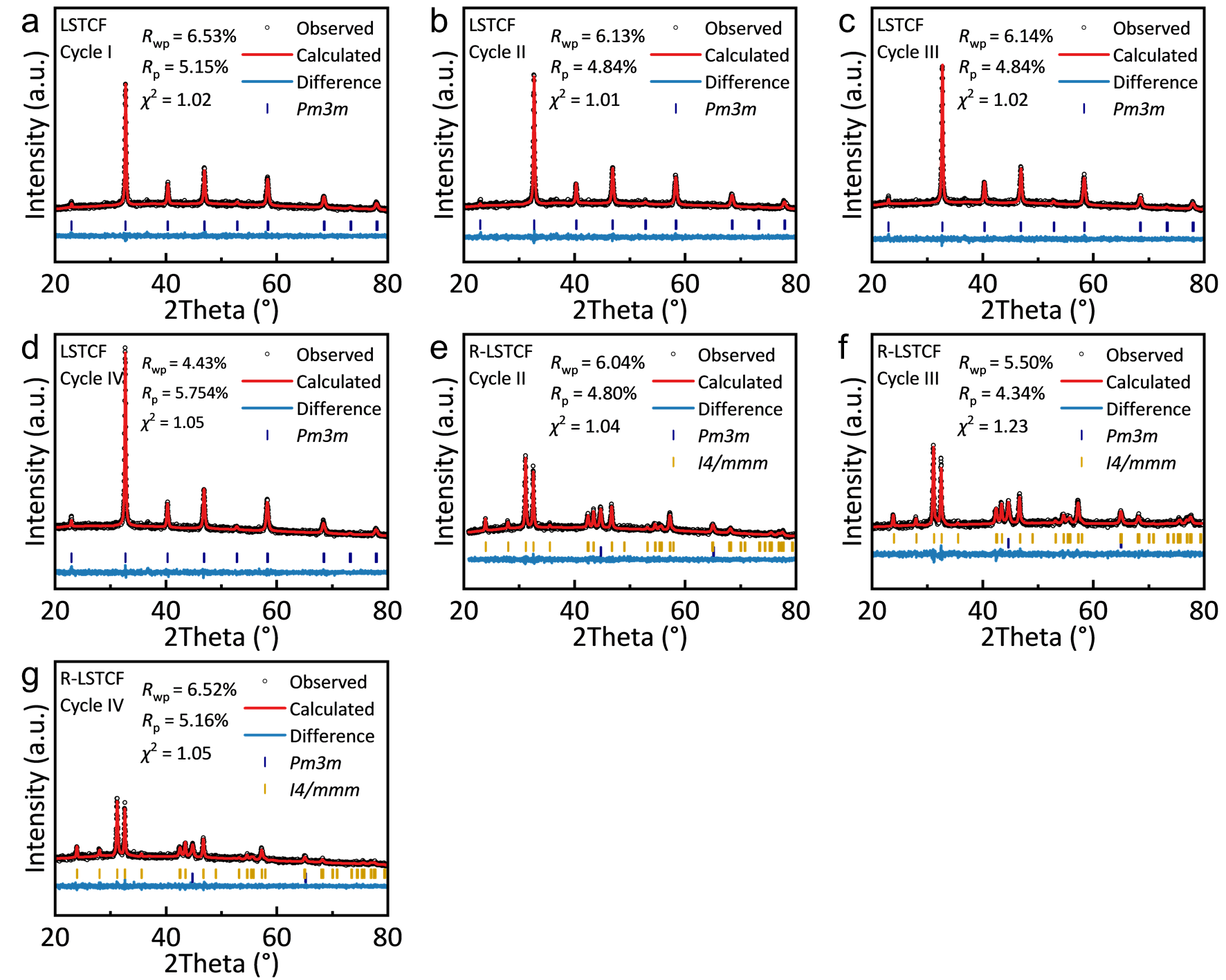


**Fig. S3** XRD refinement patterns of LSTCF after the redox cycling: **a** Cycle I LSTCF, **b** Cycle II LSTCF, **c** Cycle III LSTCF, **d** Cycle IV LSTCF, **e** Cycle II R-LSTCF, **f** Cycle III R-LSTCF, **g** Cycle IV R-LSTCF


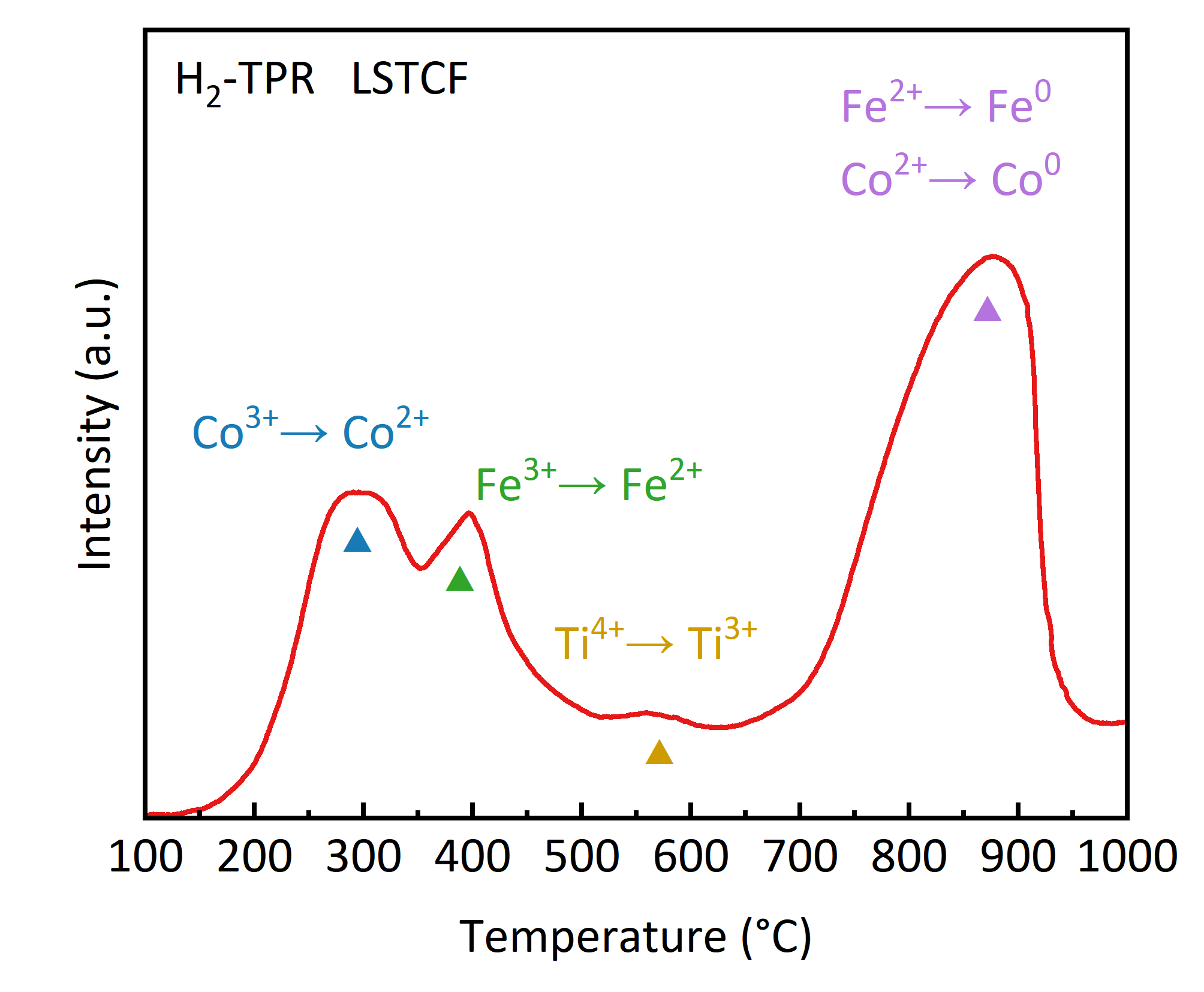


**Fig. S4** H_2_-TPR results for LSTCF


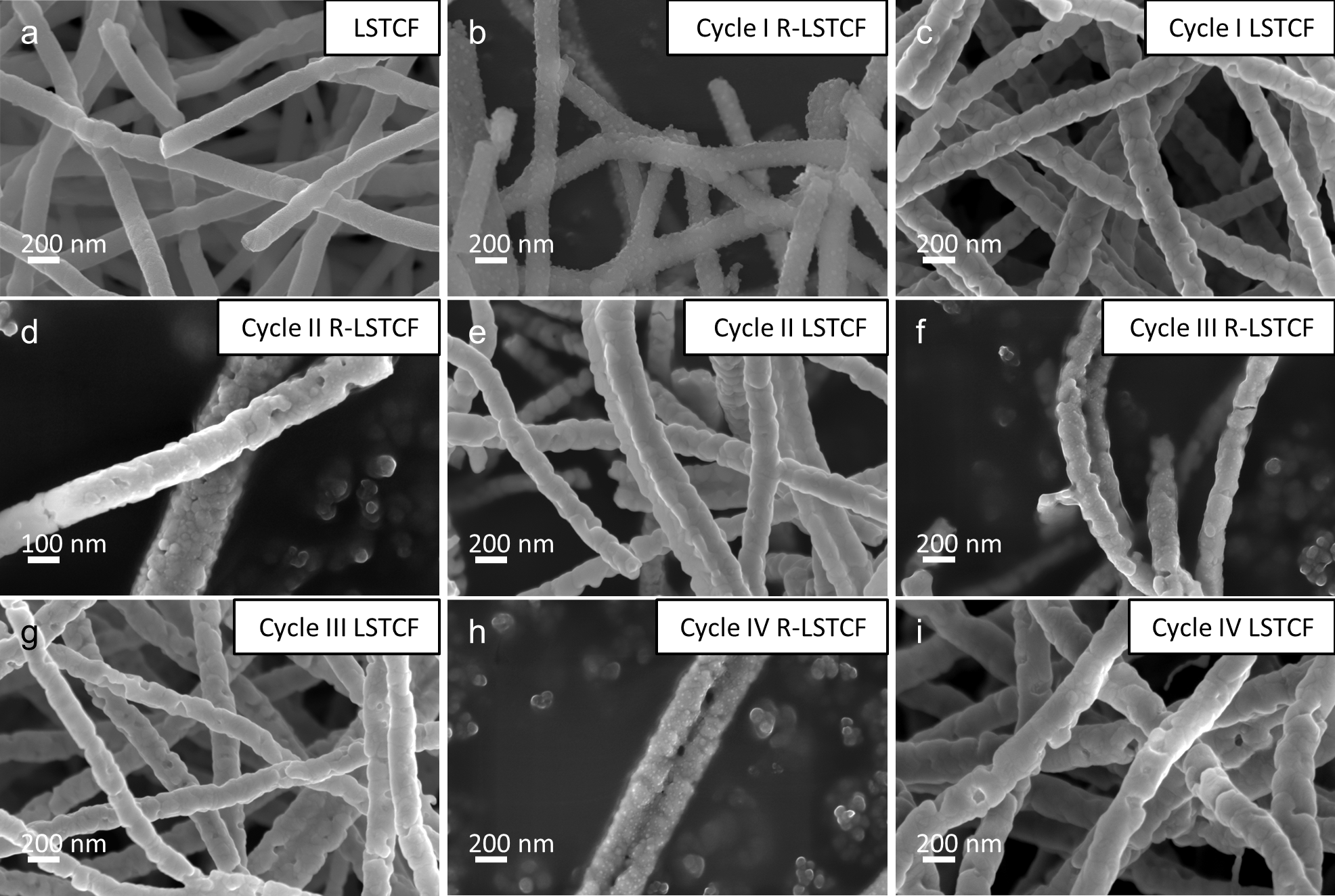


**Fig. S5** SEM images of LSTCF fibers: **a** Pristine LSTCF, **b** Cycle I R-LSTCF, **c** Cycle I LSTCF, **d** Cycle II R-LSTCF, **e** Cycle II LSTCF, **f** Cycle III R-LSTCF, **g** Cycle III LSTCF, **h** Cycle IV R-LSTCF, **i** Cycle IV LSTCF


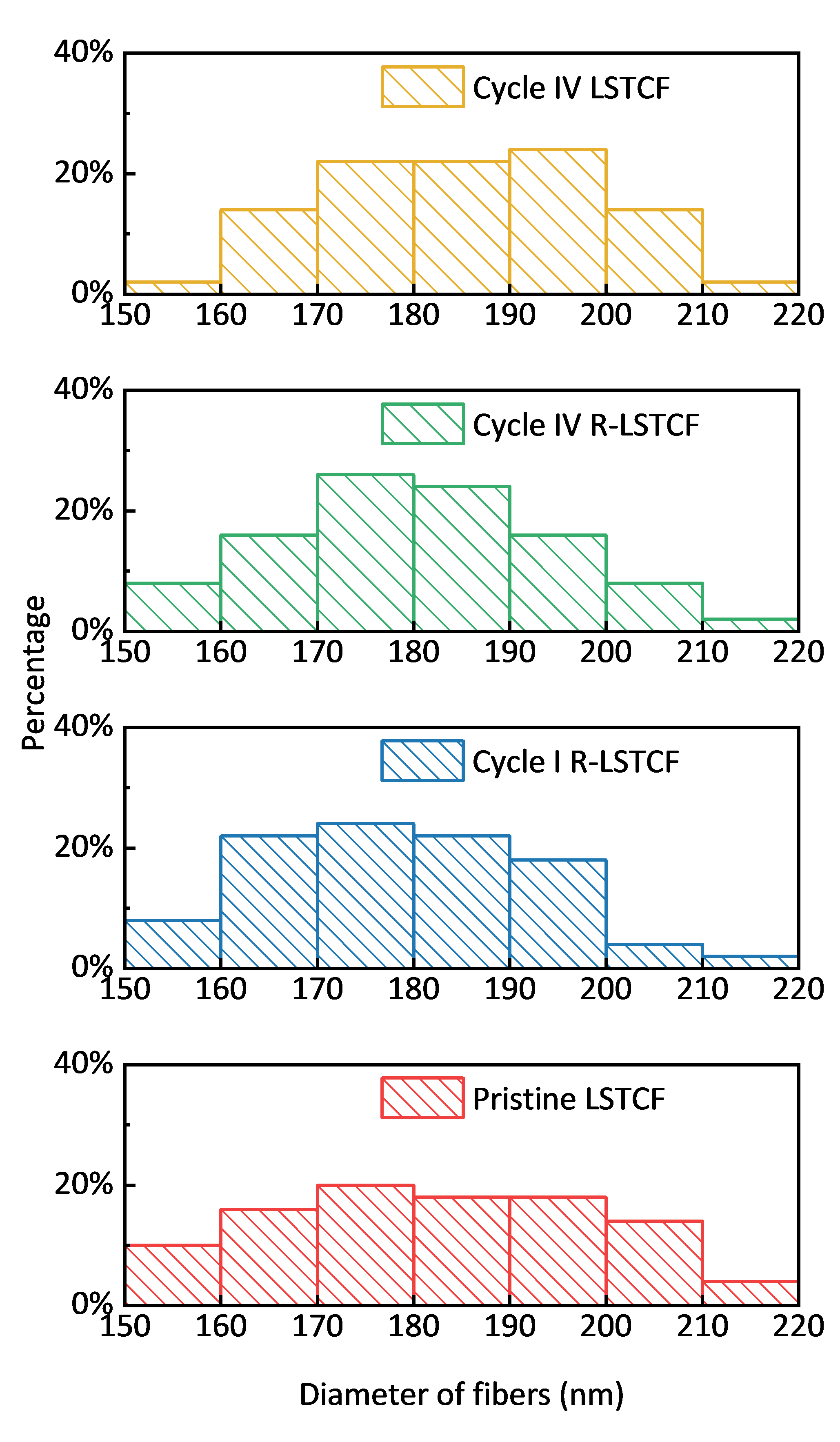


**Fig. S6** Diameter distribution of LSTCF and R-LSTCF fibers collected from SEM images by ImageJ


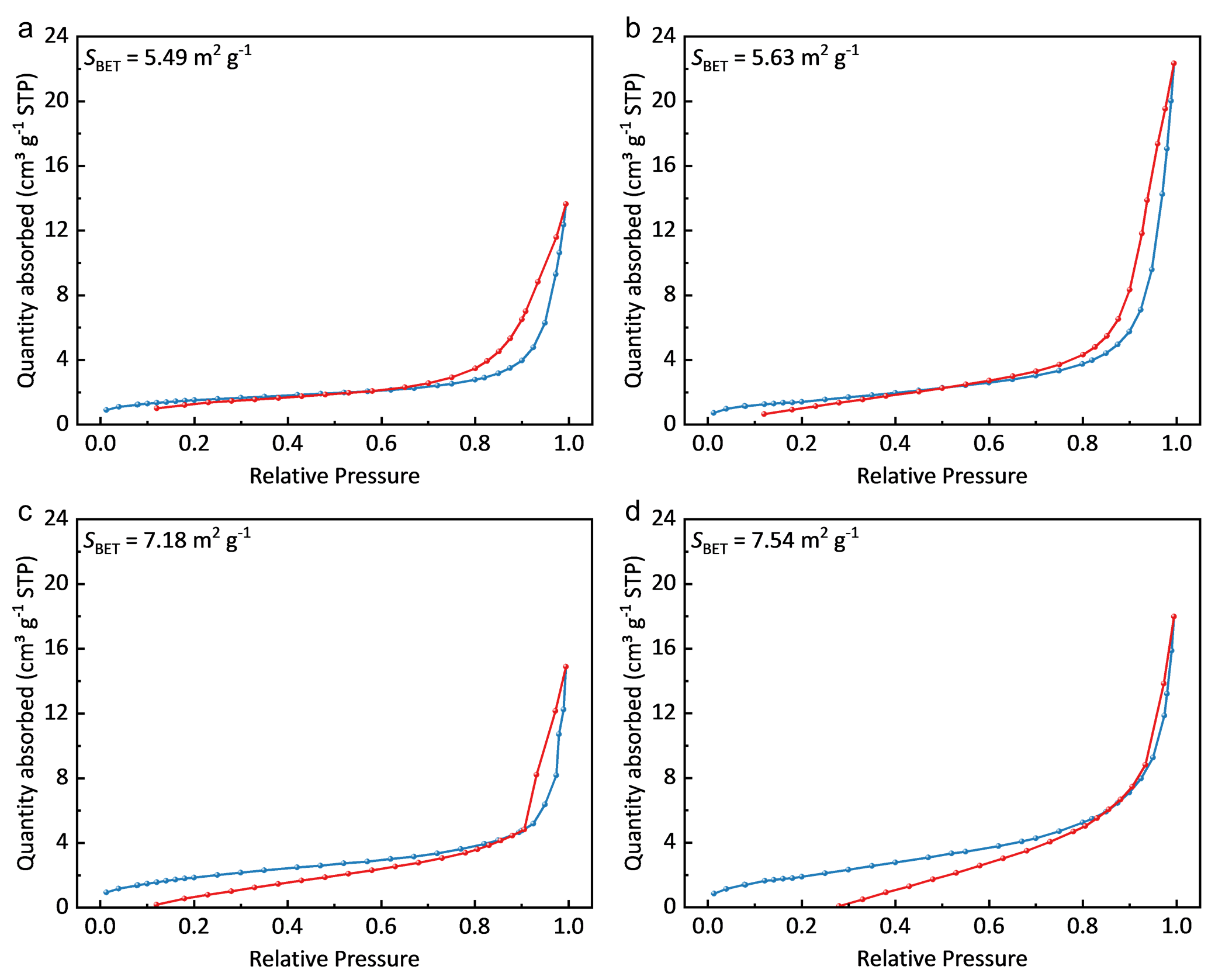


**Fig. S7** BET curves of fibers: **a** Pristine LSTCF, **b** Cycle I R-LSTCF, **c** Cycle IV LSTCF, **d** Cycle IV R-LSTCF


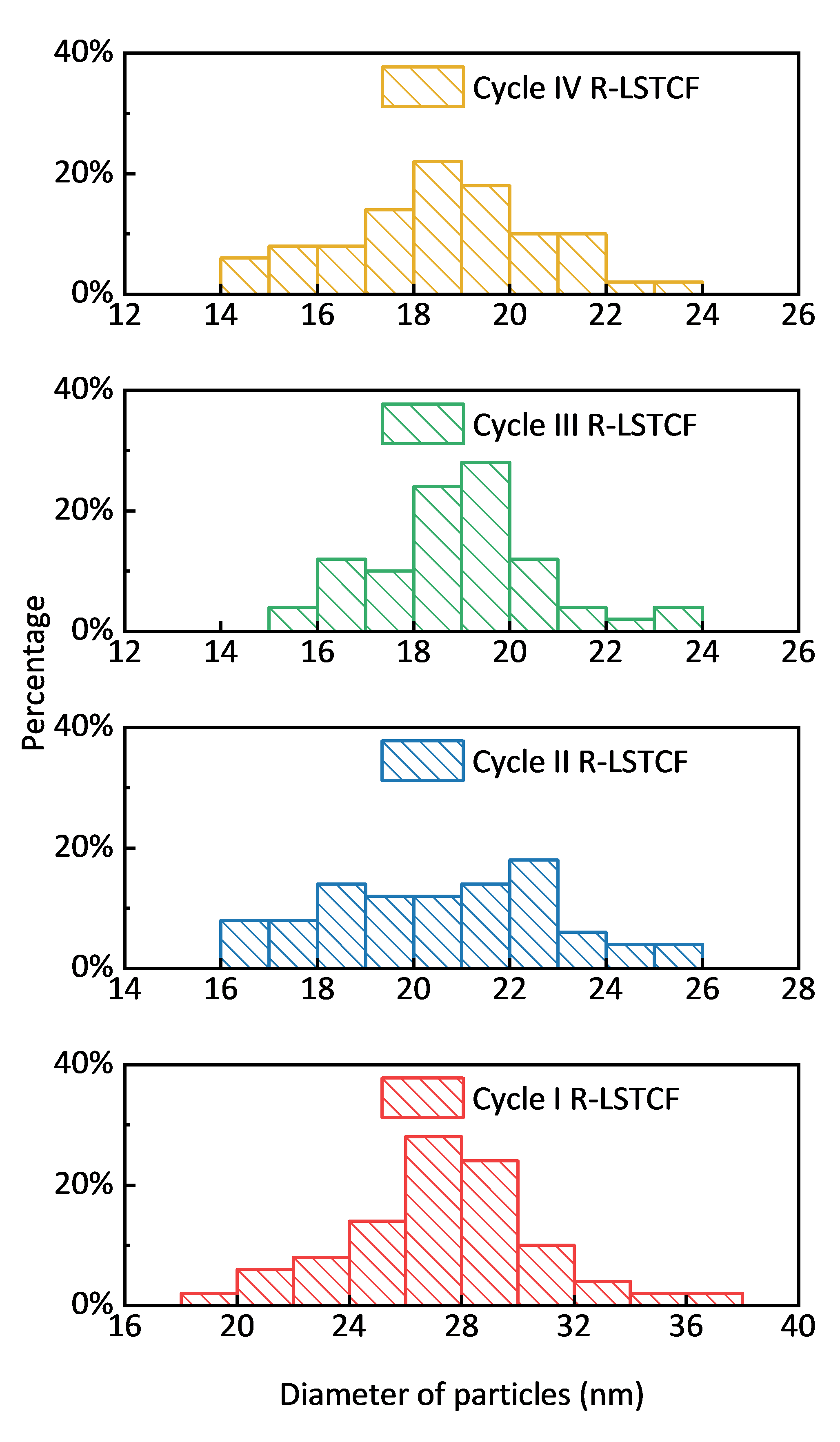


**Fig. S8** Nanoparticles diameter distributions of LSTCF and R-LSTCF collected from SEM images by ImageJ


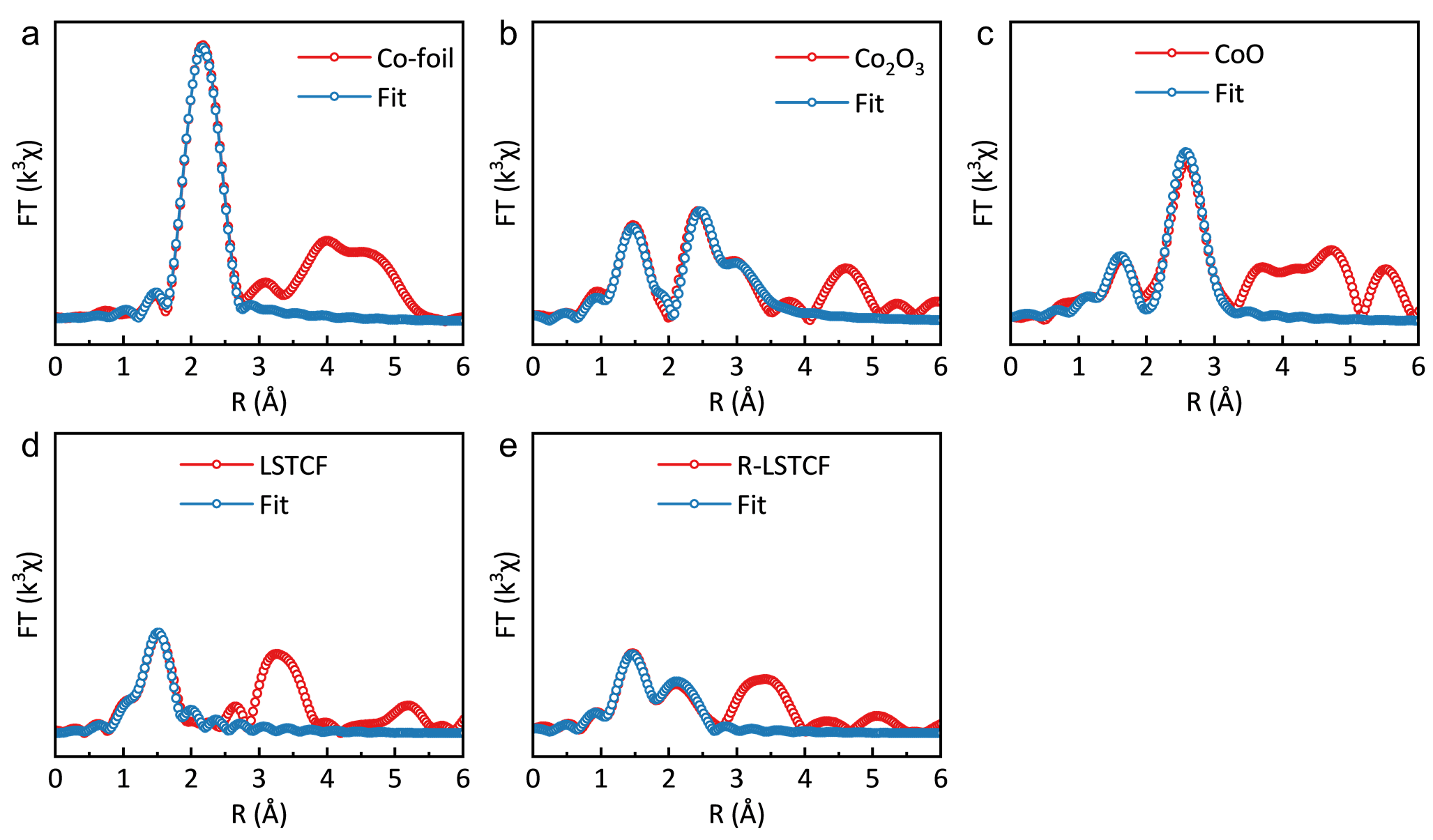


**Fig. S9** The raw and fitted Fourier transform of k^3^-weighted EXAFS spectra of Co K-edge: **a** Co-foil, **b** Co_2_O_3_, **c** CoO, **d** LSTCF, **e** R-LSTCF


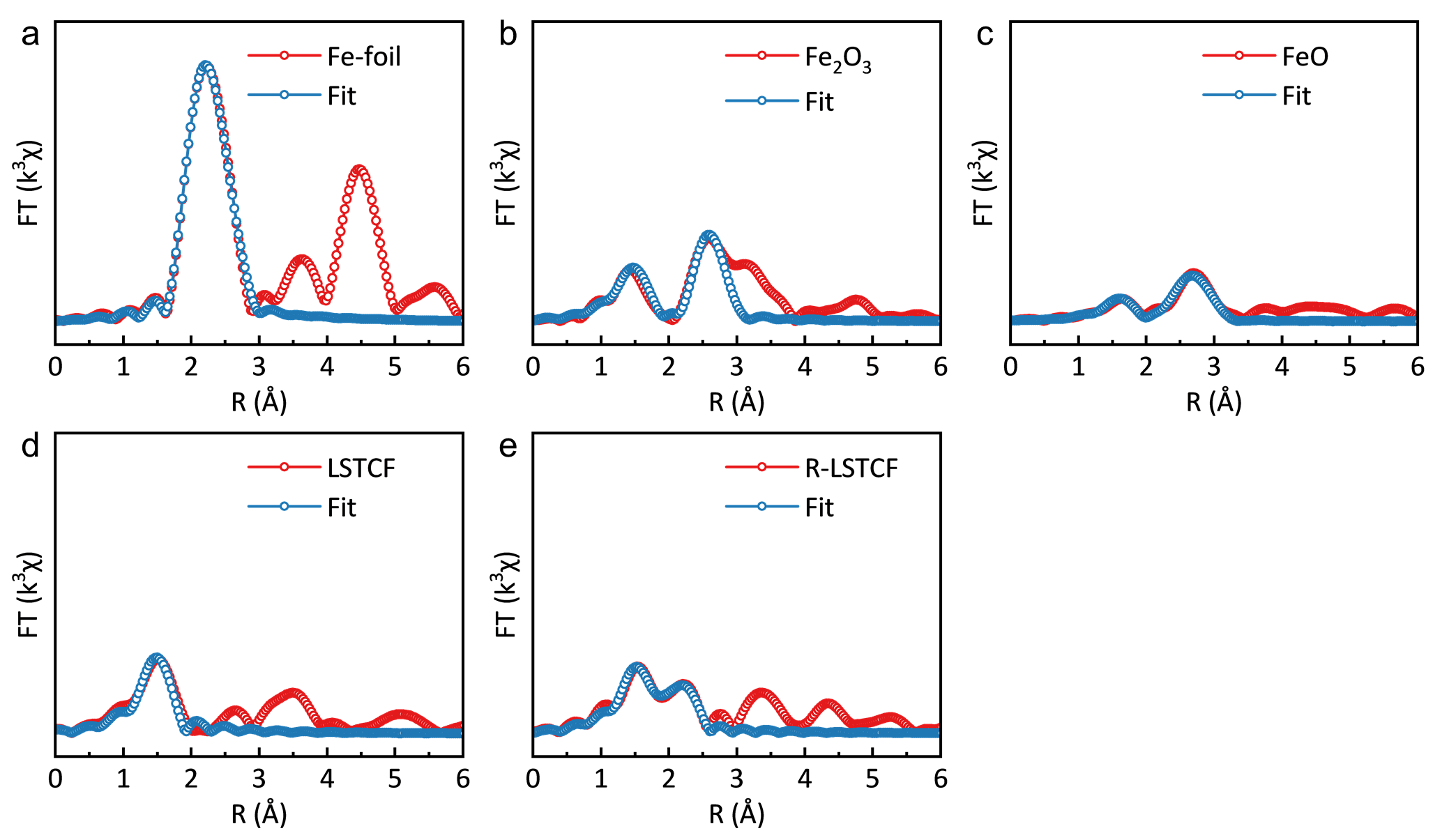


**Fig. S10** The raw and fitted Fourier transform of k^3^-weighted EXAFS spectra of Fe K-edge: **a** Fe-foil, **b** Fe_2_O_3_, **c** FeO, **d** LSTCF, **e** R-LSTCF


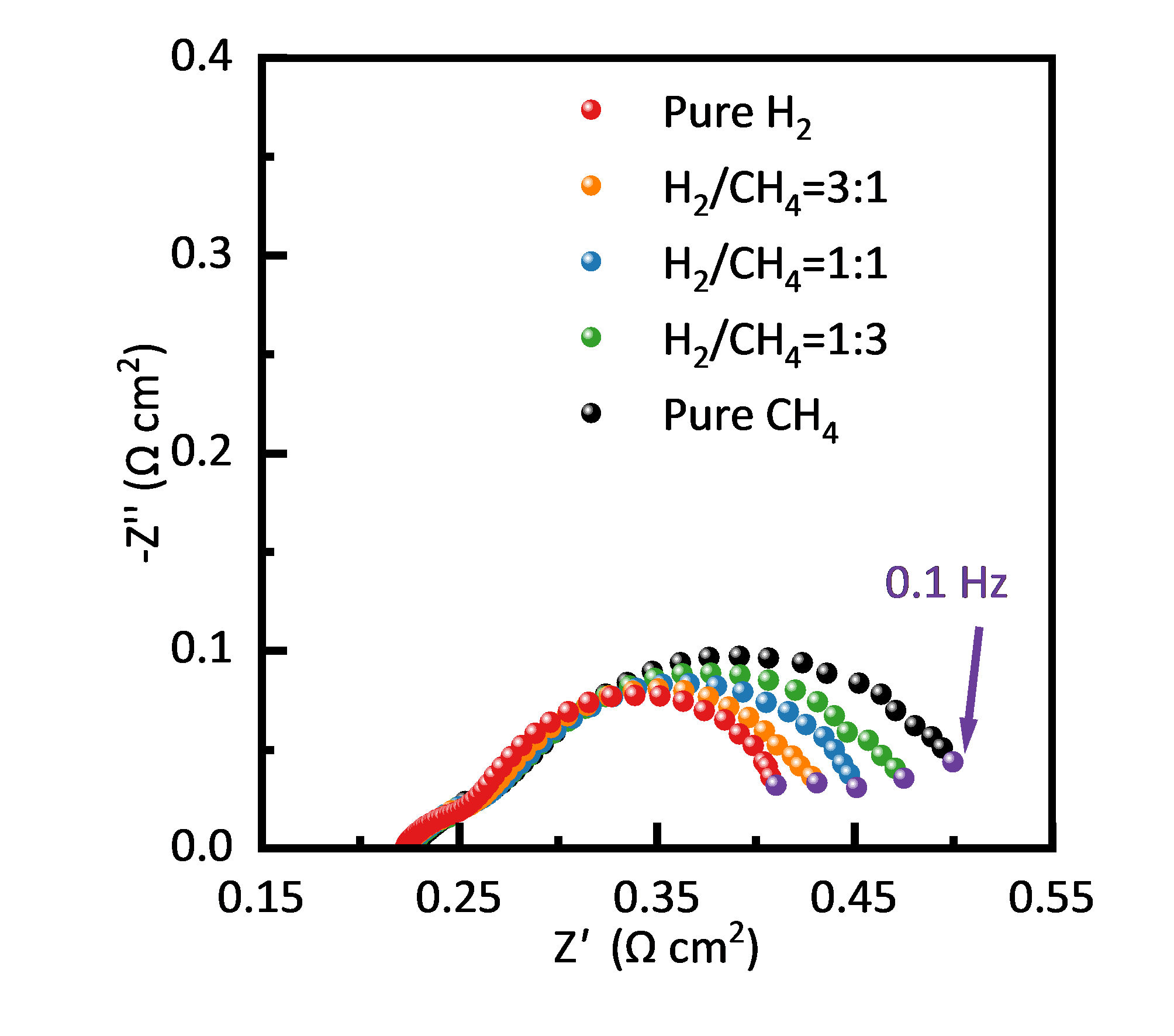


**Fig. S11** The Nyquist curves of the EIS of the single cell at 800 ℃ with different fuels under OCV conditions


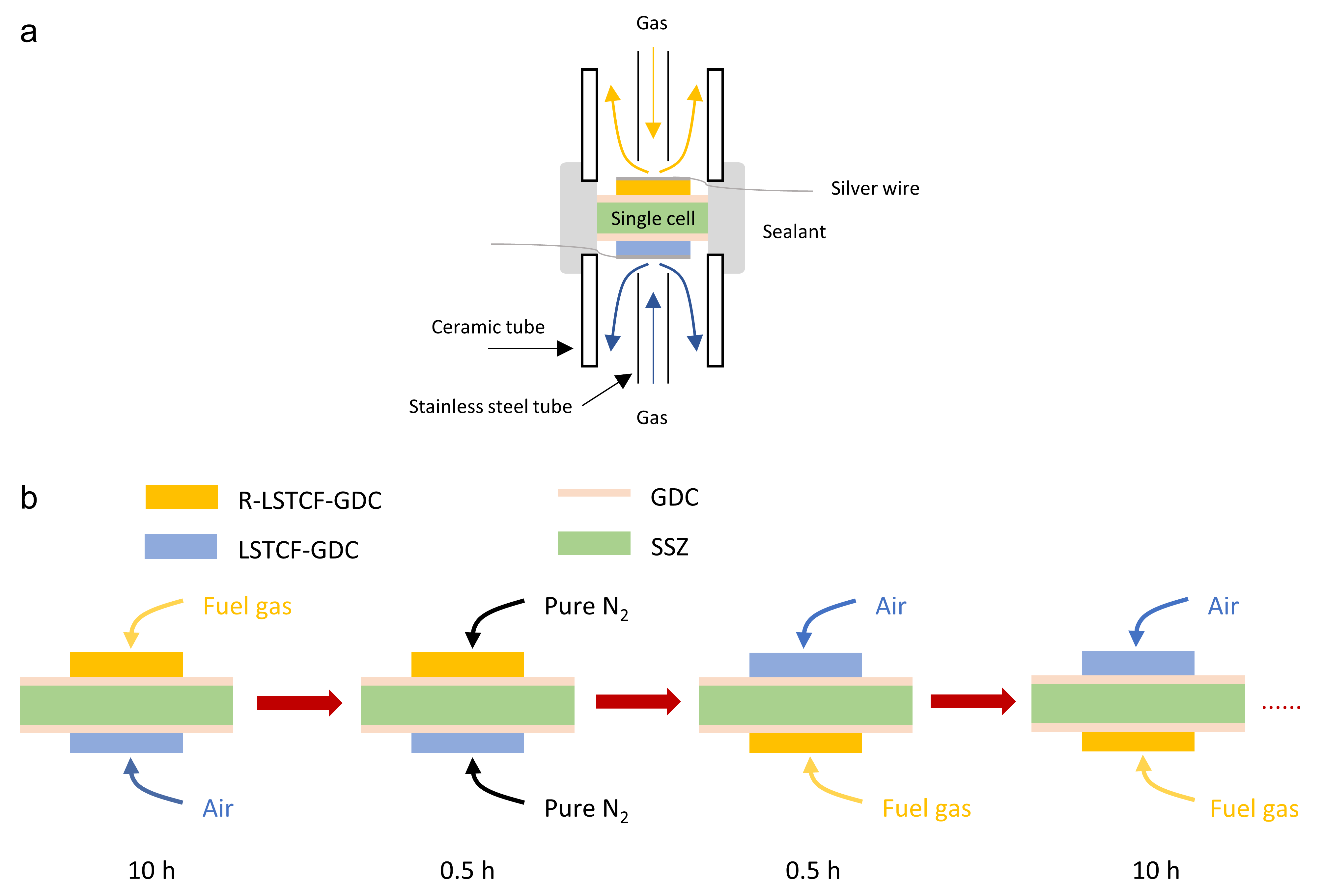


**Fig. S12** Schematic illustration of **a** the device for single cell test, **b** the reversibility test of the electrodes by switching the gases between the fuel electrode and air electrode


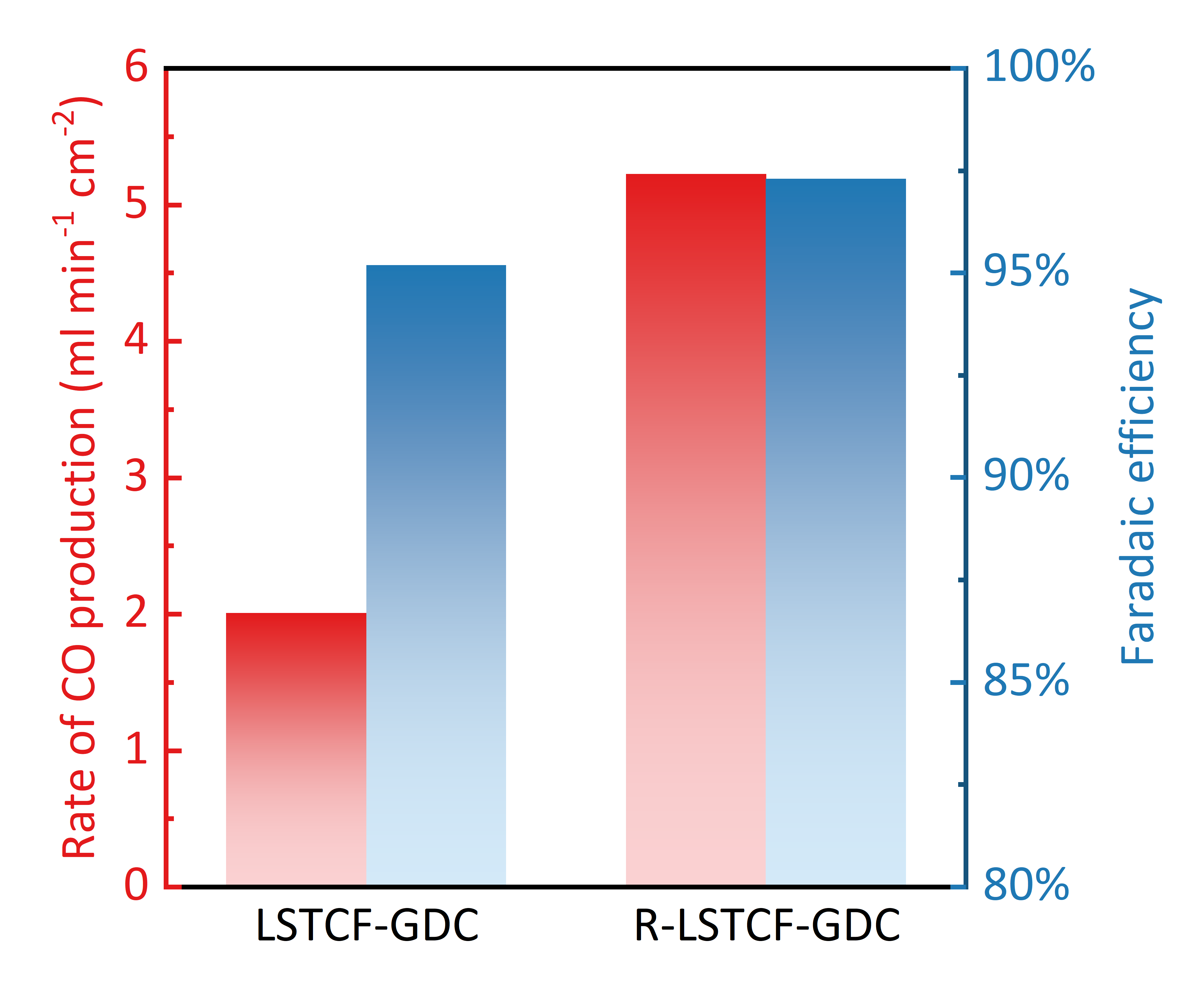


**Fig. S13** CO production rate and Faradaic efficiency of the cell for CO_2_ electrolysis


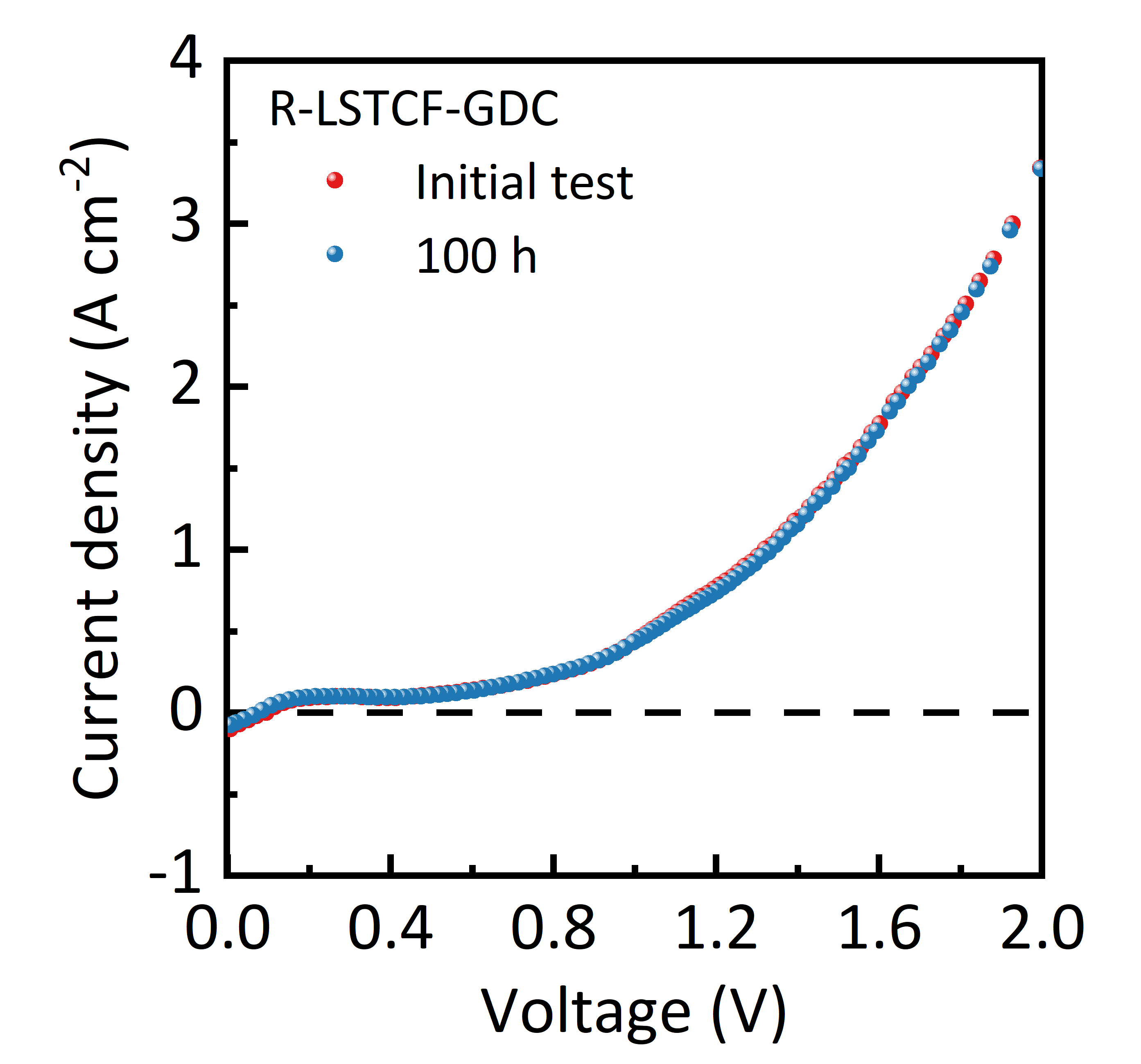


**Fig. S14** I-V curves of the R-LSTCF-GDC cell for CO_2_ electrolysis before and after the stability test

**
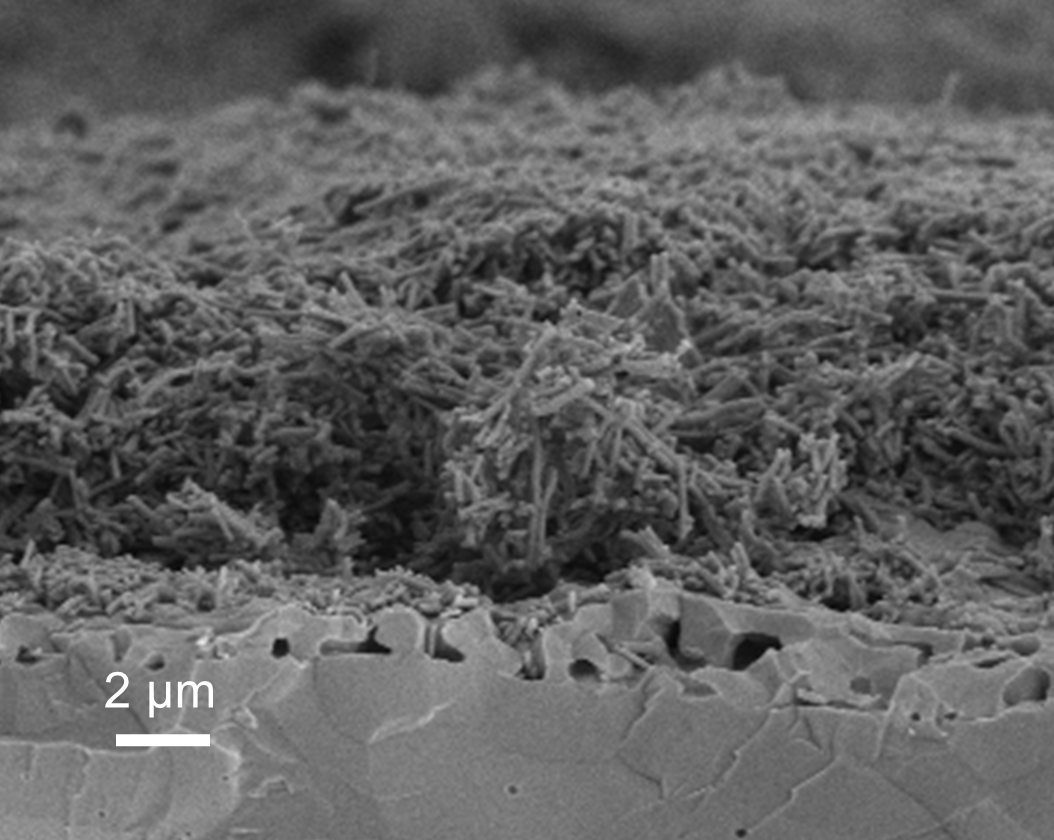
**

**Fig. S15** Cross-sectional SEM image of cell after test


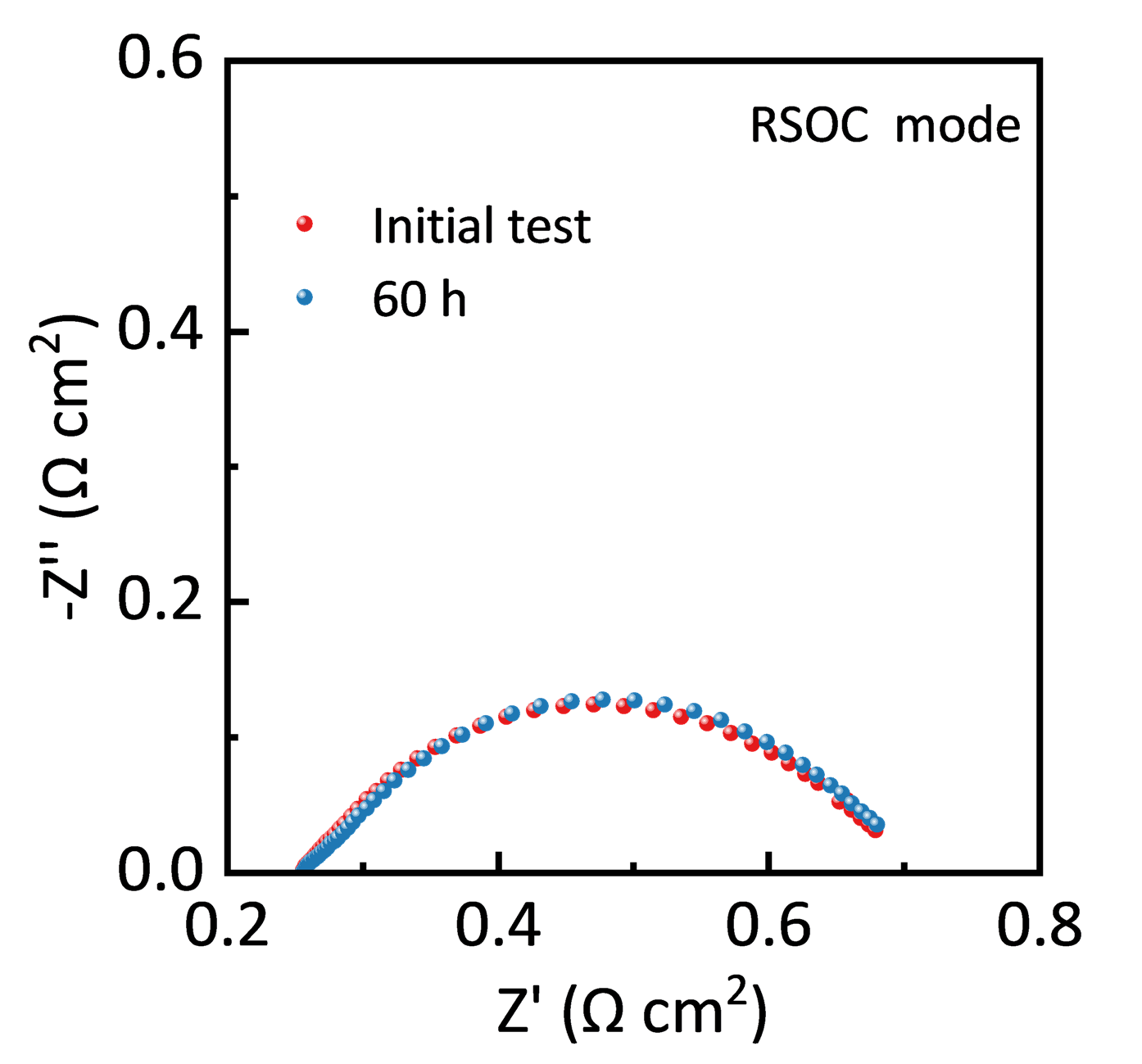


**Fig. S16** Nyquist curves of the EIS of the single cell before and after the reversible operation in the RSOC mode


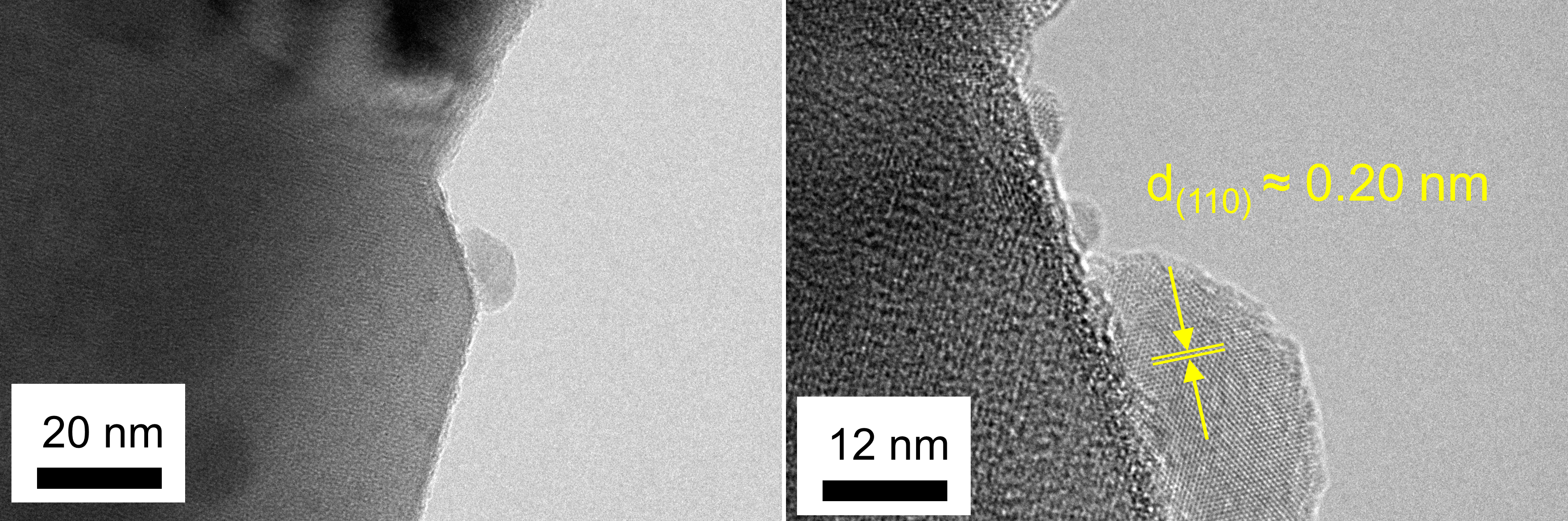


**Fig. S17** Microstructure of the electrode of the single cell after the reversible operation

**Table S1** Cell parameters of Rietveld refinements for LSTCF

| Simples | *a* (Å) (*a* = *b* = *c*) | Space group |
| --- | --- | --- |
| Pristine | 3.87097 | *Pm3m* |
| Cycle I | 3.87399 |  |
| Cycle II | 3.87383 |  |
| Cycle III | 3.87309 |  |
| Cycle IV | 3.87291 |  |

**Table S2** Rietveld refinements results for R-LSTCF

| Simples | Chemical Formula | Percentage | Space group | *a* (Å)  (*a* = *b*) | *c* (Å) |
| --- | --- | --- | --- | --- | --- |
| Cycle I | La_0.606_Sr_1.212_Ti_0.194_Co_0.105_Fe_0.701_O_4_ | 90.28% | *I4/mmm* | 3.88854 | 12.73899 |
|  | Co_3_Fe_7_ | 9.42% | *Pm3m* | 2.86640 | 2.86640 |
| Cycle II | La_0.624_Sr_1.248_Ti_0.205_Co_0.097_Fe_0.698_O_4_ | 90.61% | *I4/mmm* | 3.89304 | 12.75637 |
|  | Co_3_Fe_7_ | 9.39% | *Pm3m* | 2.87010 | 2.87010 |
| Cycle III | La_0.620_Sr_1.240_Ti_0.206_Co_0.095_Fe_0.699_O_4_ | 90.77% | *I4/mmm* | 3.89297 | 12.75283 |
|  | Co_3_Fe_7_ | 9.23% | *Pm3m* | 2.87112 | 2.87112 |
| Cycle IV | La_0.618_Sr_1.236_Ti_0.209_Co_0.095_Fe_0.696_O_4_ | 90.85% | *I4/mmm* | 3.89384 | 12.75712 |
|  | Co_3_Fe_7_ | 9.15% | *Pm3m* | 2.87222 | 2.87222 |

**Table S3** Structural parameters extracted from the Co K-edge EXAFS fitting

| Sample | Scattering pair | CN | R (Å) | σ^2^ (10^-3^ Å^2^) | ΔE_0_ (eV) | R factor |
| --- | --- | --- | --- | --- | --- | --- |
| Co-foil | Co-Co | 12.0* | 2.50 | 7.7±0.3 | 8.4±0.7 | 0.007 |
| Co_2_O_3_ | Co-O | 5.1±0.9 | 1.91 | 2.1±0.2 | -7.4±1.3 | 0.015 |
|  | Co-Co1 | 2.6±0.1 | 2.94 | 9.7±0.1 | 2.4±1.1 |  |
|  | Co-Co2 | 0.8±0.3 | 3.40 | 4.2±0.7 | -5.3±1.4 |  |
| CoO | Co-O | 5.4±0.8 | 2.11 | 7.7±0.5 | -2.0±0.4 | 0.017 |
|  | Co-Co | 9.3±0.8 | 3.00 | 8.0±1.3 | -5.7±2.1 |  |
| LSTCF | Co-O | 5.5±0.3 | 1.91 | 5.0±2.6 | -3.3±1.2 | 0.012 |
| R-LSTCF | Co-O | 4.4±0.7 | 1.91 | 3.7±0.2 | -5.9±1.1 | 0.018 |
|  | Co-Co | 1.4±0.2 | 2.50 | 9.0±0.5 | -4.8±1.4 |  |

S0^2^ is fixed as 0.7, ΔE_0_ is refined as a global parameter, |ΔE_0_| ≤ 10 eV. Data range: 3 Å ≤ k ≤ 11 Å. CN is the coordination number, R is the interatomic distance. σ^2^ is Debye-Waller factor, σ^2^ ≤ 0.01. R factor is used to value the goodness of the fitting, R factor ≤ 0.02. * This value was fixed during fitting, based on the known structure of Co.

**Table S4** Structural parameters extracted from the Fe K-edge EXAFS fitting

| Sample | Scattering pair | CN | R (Å) | σ^2^ (10^-3^ Å^2^) | ΔE_0_ (eV) | R factor |
| --- | --- | --- | --- | --- | --- | --- |
| Fe-foil | Fe-Fe1 | 8.0* | 2.46 | 4.9±1.1 | 5.6±1.6 | 0.010 |
|  | Fe-Fe2 | 6.0* | 2.85 | 3.0±0.2 | 5.3±1.0 |  |
| Fe_2_O_3_ | Fe-O | 4.6±0.4 | 2.03 | 8.5±0.2 | -6.2±2.7 | 0.014 |
|  | Fe-Fe | 10.9±1.3 | 3.00 | 7.6±1.9 | 2.0±0.8 |  |
| FeO | Fe-O | 1.9±0.3 | 2.07 | 5.8±2.1 | -2.4±0.2 | 0.008 |
|  | Fe-Fe | 9.4±0.7 | 3.09 | 6.3±2.9 | 3.4±0.4 |  |
| LSTCF | Fe-O | 5.9±0.2 | 1.95 | 5.4±0.9 | -4.6±2.8 | 0.019 |
| R-LSTCF | Fe-O | 5.5±0.7 | 1.96 | 7.0±0.5 | -4.0±0.8 | 0.008 |
|  | Fe-Fe | 2.7±0.6 | 2.50 | 7.0±2.0 | -1.3±0.4 |  |

S0^2^ is fixed as 0.7, ΔE_0_ is refined as a global parameter, |ΔE_0_| ≤ 10 eV. Data range: 3 Å ≤ k ≤ 11 Å. CN is the coordination number, R is the interatomic distance. σ^2^ is Debye-Waller factor, σ^2^ ≤ 0.01. R factor is used to value the goodness of the fitting, R factor ≤ 0.02. * This value was fixed during fitting, based on the known structure of Fe.

**Table S5** Summary of property and performance of SSZ or YSZ electrolyte-supported SOFCs.

| Fuel electrode | Electrolyte | Air electrode | Fuel gas | Peak power density (W cm^-2^) | References |
| --- | --- | --- | --- | --- | --- |
| **LSTCF-GDC*** | **SSZ**  **(~200 µm)** | **LSTCF-GDC** | **H_2_**  **(3% H_2_O)** | **0.98 (800 ℃)** | **This work** |
|  |  |  | **CH_4_**  **(3% H_2_O)** | **0.53 (800 ℃)** |  |
| La_0.52_Ca_0.28_Ni_0.06_Ti_0.94_O_3_***** | YSZ (~200 µm) | (La_0.8_Sr_0.2_)_0.95_MnO_3_ (LSM)-YSZ | H_2_ (3% H_2_O) | 0.38 (900 ℃) | [S1] |
| La_0.8_Ce_0.1_Ni_0.4_Ti_0.6_O_3_ | SSZ  (~120 µm) | LSM-SSZ | H_2_ (3% H_2_O) | 0.64 (900 ℃) | [S2] |
| **La_0.4_Sr_0.4_Ti_0.9_Ni_0.1_O_3_-GDC*** | SSZ (~250 µm) | (La_0.6_Sr_0.4_)_0.95_Co_0.2_Fe_0.8_O_3-δ_-GDC | H_2_ (3% H_2_O) | 0.85 (800 ℃) | [S3] |
| La_0.43_Sr_0.37_Cu_0.12_Ti_0.88_O_3_ | SSZ  (~90 µm) | LSM-SSZ | H_2_ (3% H_2_O) | 1.04 (800 ℃) | [S4] |
| La_0.43_Sr_0.37_Fe_0.09_Cu_0.03_Ti_0.88_O_3_ | SSZ  (~85 µm) | LSM-SSZ | H_2_ (3% H_2_O) | 0.68 (800 ℃) | [S5] |
| **(La_0.6_Sr_1.4_)_0.95_Mn_0.9_**  **Cu_0.1_O_4_-SSZ** | SSZ  (~185 µm) | (La_0.6_Sr_1.4_)_0.95_Mn_0.9_Cu_0.1_O_4_-SSZ | H_2_ | 0.62 (750 ℃) | [S6] |
| La_0.43_Ca_0.37_Ni_0.03_Fe_0.03_Ti_0.94_O_3_ | SSZ  (~250 µm) | LSM-SSZ | H_2_ | 0.43 (900 ℃) | [S7] |
| La_0.43_Ca_0.37_Ni_0.06_Ti_0.94_O_3_ | SSZ  (~150 µm) | LSM-YSZ | H_2_ (25% N_2_+25% H_2_O) | 0.29 (850 ℃) | [S8] |
| **NiO-YSZ** |  |  |  | 0.26 (850 ℃) |  |
| **La_0.9_Ca_0.1_Fe_0.9_Nb_0.1_O_3_-SDC** | SSZ  (~60 µm) | La_0.9_Ca_0.1_Fe_0.9_Nb_0.1_O_3_-SDC | H_2_ | 0.53 (850 ℃) | [S9] |
| PrBaMn_2_O_5+δ_-Pr_6_O_11_ | YSZ | PrBaMn_2_O_5+δ_-Pr_6_O_11_ | Wet H_2_ | 0.42 (800 ℃) | [S10] |
| **La_0.3_Sr_0.6_Ni_0.1_Ti_0.9_O_3_-GDC*** | SSZ (~200 µm) | La_0.2_Sr_0.8_MnO_3_-GDC | H_2_ (3% H_2_O) | 1.16 (800 ℃) | [S11] |

Composite fuel electrodes are shown in bold. * Fiber electrodes.

**Table S6** Summary of property and performance of SSZ or YSZ electrolyte-supported SOEC for CO_2_ electrolysis

| Fuel electrode | Electrolyte | Air electrode | Fuel gas | Current density at 1.4 V (A cm^-2^) | References |
| --- | --- | --- | --- | --- | --- |
| **LSTCF-GDC*** | **SSZ**  **(~200 µm)** | **LSTCF-GDC** | **CO_2_** | **0.55 (800 ℃)** | **This work** |
| **R-LSTCF-GDC*** |  | **LSTCF-GDC** |  | **1.18 (800 ℃)** |  |
| La_0.3_Ca_0.6_Ni_0.05_Mn_0.08_Ti_0.9_O_3_ | SSZ  (~150 µm) | LSM-SSZ | CO_2_ (3% H_2_O) | 0.57 (900 ℃) | [S12] |
|  |  |  | CO_2_ (19.4% H_2_+3% H_2_O) | 0.91 (900 ℃) |  |
| (La_4_Sr_4_)_0.9_Ti_7.2_Ni_0.8_O_26_ | YSZ  (~500 µm) | La_0.4_Sr_0.6_Co_0.2_Fe_0.8_O_3−δ_(LSCF) | CO_2_ (15% CO) | 0.63 (800 ℃) | [S13] |
| **(La_0.2_Sr_0.8_)_0.95_Ti_0.85_Mn_0.1_Ni_0.05_O_3+δ_-SDC** | YSZ  (~500 µm) | LSM-SDC | CO_2_ | 0.36 (800 ℃) | [S14] |
| **Sr_2_FeCo_0.2_Ni_0.2_Mn_0.1_Mo_0.5_O_6−δ_- Ce_0.8_Gd_0.2_O_2_ (CGO)** | SSZ  (~150 µm) | LSCF | CO_2_ (75% Ar) | 0.32 (800 ℃) | [S15] |
| La_0.43_Ca_0.37_Ni_0.03_Fe_0.03_Ti_0.94_O_3_ | SSZ  (~250 µm) | LSM-SSZ | CO_2_ | 0.36 (900 ℃) | [S7] |
| **La_0.75_Sr_0.2_Ca_0.05_Cr_0.5_Mn_0.5_O_3_-GDC** | SSZ  (~200 µm) | La_0.8_Sr_0.2_MnO_3-δ_-GDC | CO_2_ | 0.35 (900 ℃) | [S16] |
| **Pr_0.25_(La_0.75_Sr_0.25_)_0.75_Cr_0.5_Mn_0.5_O_3-δ_-GDC** | SSZ  (~80 µm) | LSCF-GDC | CO_2_ | 0.56 (800 ℃) | [S17] |
| La_0.43_Ca_0.37_Ni_0.06_Ti_0.94_O_3_ | SSZ  (~150 µm) | LSM-YSZ | CO_2_ (50% H_2_+25% H_2_O) | 0.68 (850 ℃) | [S8] |
| **NiO-YSZ** |  |  |  | 0.49 (850 ℃) |  |
| **La_0.6_Ca_0.4_Fe_0.8_Ni_0.2_O_3−δ_-GDC** | YSZ  (~300 µm) | La_0.6_Ca_0.4_Fe_0.8_Ni_0.2_O_3−δ_-GDC | CO_2_ | 0.65 (800 ℃) | [S18] |
| La_0.52_Ca_0.28_Ni_0.04_Fe_0.04_Ti_0.92_O_3_* | YSZ  (~200 µm) | LSM-YSZ | CO_2_ | 0.45 (900 ℃) | [S19] |
| **Pd-La_0.5_Sr_0.5_FeO_3-δ_-SDC** | YSZ  (~500 µm) | LSM-YSZ | CO_2_ (5% N_2_) | 0.44 (800 ℃) | [S20] |
| Fe-Sr_2_Fe_1.3_Ni_0.2_Mo_0.5_O_6-δ_ | YSZ  (~230 µm) | LSCF-GDC | CO_2_ | 0.55 (800 ℃) | [S21] |
| DP-Fe-Sr_2_Fe_1.2_Ni_0.3_Mo_0.5_O_6-δ_ | YSZ  (250-300µm) | LSCF-GDC | CO_2_ | 1.04 (850 ℃) | [S22] |
| **La_0.5_Sr_0.5_Fe_0.95_V_0.05_O_3-δ_-GDC** | YSZ  (~500µm) | LSM-YSZ | CO_2_ (5% N_2_) | 0.46 (800 ℃) | [S23] |

Composite fuel electrodes are shown in bold. * Fiber electrodes.

**Supplementary References**

1. M. Xu, R. Cao, S. Wu, J. Lee, D. Chen et al., Nanoparticle exsolution via electrochemical switching in perovskite fibers for solid oxide fuel cell electrodes. J. Mater. Chem. A **11**, 13007-13015 (2023). <https://doi.org/10.1039/d3ta00535f>
2. S. He, M. Li, J. Hui, X. Yue, In-situ construction of ceria-metal/titanate heterostructure with controllable architectures for efficient fuel electrochemical conversion. Appl. Catal. B Environ. **298**, 120588 (2021). <https://doi.org/10.1016/j.apcatb.2021.120588>
3. J. Yang, J. Zhou, Z. Liu, Y. Sun, C. Yin et al., Exploring heterogeneous phases in highly A-site-deficient titanate with Ni exsolution. J. Power Sources **580**, 233369 (2023). <https://doi.org/10.1016/j.jpowsour.2023.233369>
4. S. Jo, H. G. Jeong, Y. H. Kim, D. Neagu, J.-h. Myung, Stability and activity controls of Cu nanoparticles for high-performance solid oxide fuel cells. Appl. Catal. B Environ. **285**, 119828 (2021). <https://doi.org/10.1016/j.apcatb.2020.119828>
5. S. Jo, Y. Han Kim, H. Jeong, C.-h. Park, B.-R. Won et al., Exsolution of phase-separated nanoparticles via trigger effect toward reversible solid oxide cell. Appl. Energy **323**, 119615 (2022). <https://doi.org/10.1016/j.apenergy.2022.119615>
6. J. Wang, J. Zhou, J. Yang, Z. Zong, L. Fu, et al., Nanoscale architecture of (La_0.6_Sr_1.4_)_0.95_Mn_0.9_B_0.1_O_4_ (B=Co, Ni, Cu) Ruddlesden–Popper oxides as efficient and durable catalysts for symmetrical solid oxide fuel cells. Renewable Energy **157,** 840-850 (2020). <https://doi.org/10.1016/j.renene.2020.05.014>
7. M. Chanthanumataporn, J. Hui, X. Yue, K. Kakinuma, J. T. S. Irvine et al., Electrical reduction of perovskite electrodes for accelerating exsolution of nanoparticles. Electrochim. Acta **306**, 159-166 (2019). <https://doi.org/10.1016/j.electacta.2019.03.126>
8. V. Kyriakou, D. Neagu, E. I. Papaioannou, I. S. Metcalfe, M. C. M. van de Sanden et al., Co-electrolysis of H_2_O and CO_2_ on exsolved Ni nanoparticles for efficient syngas generation at controllable H_2_/CO ratios. Appl. Catal. B Environ. **258**, 117950 (2019). <https://doi.org/10.1016/j.apcatb.2019.117950>
9. X. Kong, X. Zhou, Y. Tian, X. Wu, J. Zhang et al., Niobium doped lanthanum calcium ferrite perovskite as a novel electrode material for symmetrical solid oxide fuel cells. J. Power Sources **326**, 35-42 (2016). <https://doi.org/10.1016/j.jpowsour.2016.06.111>
10. Y. Gu, Y. Zhang, Y. Zheng, H. Chen, L. Ge et al., PrBaMn_2_O_5+δ_ with praseodymium oxide nano-catalyst as electrode for symmetrical solid oxide fuel cells. Appl. Catal. B Environ. **257**, 117868 (2019). <https://doi.org/10.1016/j.apcatb.2019.117868>
11. J. Zhou, J. Yang, Z. Zong, L. Fu, Z. Lian et al., A mesoporous catalytic fiber architecture decorated by exsolved nanoparticles for reversible solid oxide cells. J. Power Sources **468**, 228349 (2020). <https://doi.org/10.1016/j.jpowsour.2020.228349>
12. N. Zhang, A. Naden, L. Zhang, X. Yang, P. Connor et al., Enhanced CO_2_ electrolysis through Mn substitution coupled with Ni exsolution in lanthanum calcium titanate electrodes. Adv. Mater. **36**, 2308481 (2024). <https://doi.org/10.1002/adma.202308481>
13. Z. Liu, J. Zhou, Y. Sun, X. Yue, J. Yang et al., Tuning exsolution of nanoparticles in defect engineered layered perovskite oxides for efficient CO_2_ electrolysis. J. Energy Chem. **84**, 219-227 (2023). <https://doi.org/10.1016/j.jechem.2023.05.033>
14. L. Ye, M. Zhang, P. Huang, G. Guo, M. Hong et al., Enhancing CO_2_ electrolysis through synergistic control of non-stoichiometry and doping to tune cathode surface structures. Nat. Commun. **8**, 14785 (2017). <https://doi.org/10.1038/ncomms14785>
15. A. López-García, L. Almar, S. Escolástico, A. B. Hungría, A. J. Carrillo et al., Tuning ternary alloyed nanoparticle composition and morphology by exsolution in double perovskite electrodes for CO_2_ electrolysis. ACS Appl. Energy Mater. **5**(11), 13269-13283 (2022). <https://doi.org/10.1021/acsaem.2c01829>
16. B. Qian, C. Liu, S. Wang, B. Yin, Y. Zheng et al., Ca-doped La_0.75_Sr_0.25_Cr_0.5_Mn_0.5_O_3_ cathode with enhanced CO_2_ electrocatalytic performance for high-temperature solid oxide electrolysis cells. Int. J. Hydrogen Energy **46**, 33349-33359 (2021). <https://doi.org/10.1016/j.ijhydene.2021.07.174>
17. Z. Pan, H. Shi, S. Wang, H. Jiang, Y. Zheng, Highly active and stable A-site Pr-doped LaSrCrMnO-based fuel electrode for direct CO_2_ solid oxide electrolyzer cells. Int. J. Hydrogen Energy **45**, 14648-14659 (2020). <https://doi.org/10.1016/j.ijhydene.2020.03.224>
18. Y. Tian, L. Zhang, Y. Liu, L. Jia, J. Yang et al., A self-recovering robust electrode for highly efficient CO_2_ electrolysis in symmetrical solid oxide electrolysis cells. J. Mater. Chem. A **7**, 6395-6400 (2019). <https://doi.org/10.1039/C9TA00643E>
19. M. Xu, C. Liu, A. B. Naden, H. Früchtl, M. Bühl et al., Electrochemical activation applied to perovskite titanate fibers to yield supported alloy nanoparticles for electrocatalytic application. Small **19**, 2204682 (2023). <https://doi.org/10.1002/smll.202204682>
20. Y. Zhou, L. Lin, Y. Song, X. Zhang, H. Lv et al., Pd single site-anchored perovskite cathode for CO_2_ electrolysis in solid oxide electrolysis cells. Nano Energy **71**, 104598 (2020). <https://doi.org/10.1016/j.nanoen.2020.104598>
21. B.-W. Zhang, M.-N. Zhu, M.-R. Gao, X. Xi, N. Duan et al., Boosting the stability of perovskites with exsolved nanoparticles by B-site supplement mechanism. Nat. Commun. **13**, 4618 (2022). <https://doi.org/10.1038/s41467-022-32393-y>
22. B.-W. Zhang, M.-N. Zhu, M.-R. Gao, J. Chen, X. Xi et al., Phase transition engineering of host perovskite toward optimal exsolution-facilitated catalysts for carbon dioxide electrolysis. Angew. Chem., Int. Ed. **62**, e202305552 (2023). <https://doi.org/10.1002/anie.202305552>
23. Y. Zhou, Z. Zhou, Y. Song, X. Zhang, F. Guan et al., Enhancing CO_2_ electrolysis performance with vanadium-doped perovskite cathode in solid oxide electrolysis cell. Nano Energy **50**, 43-51 (2018). <https://doi.org/10.1016/j.nanoen.2018.04.054>
